# Combination of long-read and short-read sequencing provides comprehensive transcriptome and new insight for *Chrysanthemum morifolium* ray-floret colorization

**DOI:** 10.1038/s41598-022-22589-z

**Published:** 2022-10-25

**Authors:** Mitsuko Kishi-Kaboshi, Tsuyoshi Tanaka, Katsutomo Sasaki, Naonobu Noda, Ryutaro Aida

**Affiliations:** 1 Institute of Vegetable and Floriculture Science, National Agriculture and Food Research Organization (NARO), Fujimoto 2-1, Tsukuba, Ibaraki 305-0852 Japan; 2 Research Center for Advanced Analysis, National Agriculture and Food Research Organization (NARO), Kannondai 2-1-2, Tsukuba, Ibaraki 305-8518 Japan; 3 Present Address: Institute of Crop Science, National Agriculture and Food Research Organization (NARO), Kannondai 2-1-2, Tsukuba, Ibaraki 305-8518 Japan

**Keywords:** RNA sequencing, Plant biotechnology

## Abstract

*Chrysanthemum morifolium* is one of the most popular ornamental plants globally. Owing to its large and complex genome (around 10 Gb, segmental hexaploid), it has been difficult to obtain comprehensive transcriptome, which will promote to perform new breeding technique, such as genome editing, in *C. morifolium*. In this study, we used single-molecule real-time (SMRT) sequencing and RNA-seq technologies, combined them with an error-correcting process, and obtained high-coverage ray-floret transcriptome. The SMRT-seq data increased the ratio of long mRNAs containing complete open-reading frames, and the combined dataset provided a more complete transcriptomic data than those produced from either SMRT-seq or RNA-seq-derived transcripts. We finally obtained ‘Sei Arabella’ transcripts containing 928,645 non-redundant mRNA, which showed 96.6% Benchmarking Universal Single-Copy Orthologs (BUSCO) score. We also validated the reliability of the dataset by analyzing a mapping rate, annotation and transcript expression. Using the dataset, we searched anthocyanin biosynthesis gene orthologs and performed a qRT-PCR experiment to assess the usability of the dataset. The assessment of the dataset and the following analysis indicated that our dataset is reliable and useful for molecular biology. The combination of sequencing methods provided genetic information and a way to analyze the complicated *C. morifolium* transcriptome*.*

## Introduction

*Chrysanthemum morifolium* is one of the most economically valuable flowering plants globally. To generate attractive and productive *C. morifolium* cultivars, breeding program has been performed annually. Flower color is an important economic trait because it greatly influences consumer behavior. *C. morifolium* is segmental hexaploid (2n = 6× = 54), self-incompatible^1^, and has a large genome (12–24 Gb, https://www.asteraceaegenomesize.com/#^2^, and 7.93 Gb in ‘Sei Marine’^3^). In addition, each cultivar has its own unique set of chromosomes (ranging from 47 to 63)^4^. Therefore, it has been difficult to apply genetic analysis and modern breeding technique on *C. morifolium.* Genome editing is a hopeful method to modify specific traits. To apply genome editing in *C. morifolium,* we had performed genome editing using transgenic *C. morifolium*^5^*.* To perform genome editing on agronomical traits, target gene information is necessary. It is also essential to obtain high-coverage transcriptome data to collect the genomic sequences for genome editing experiment promptly. Genomic data of diploid chrysanthemum species, *C. nankingense*^6^ and *C. seticuspe*^3^, provided fundamental genetic information of chrysanthemum species. In contrast, the ancestors of *C. morifolium* are different among cultivars, and at least seven species are considered as independent paternal ancestors, whereas the maternal ancestor is believed to be extinct^7^. Therefore, exact sequence information of *C. morifolium* is desired. Analyzing the *C. morifolium* transcriptome is a realistic way to obtain genetic information, which directly affects visible traits, including floret pigmentation. It is expected that *C. morifolium* transcripts represent the complex transcription of genes, including numerous homeologs, paralogs, and transcripts arising from alternative splicing.

Recently, several transcriptomic analyses of *C. morifolium* have been reported. Using the Illumina RNA-seq system, 63,854–400,234 de novo assembled transcripts with mean lengths of 719–1030 bp were reported^8–13^. Using a pyrosequencing system, 16,769 contigs from leaves^14^ and 213,204 contigs from floral organs, stems, and leaves^15^ were reported. The application of PacBio single-molecule real-time (SMRT) sequencing (SMRT-seq) revealed 89,477 and 130,097 transcripts in *C. morifolium*^16,17^. However, there was little information related to data quality, especially about the existence of conserved gene sets or the mapping rate of short reads used for expression analysis. De novo assembled transcriptome data containing 199,754 unique sequences showed about 74% mapping rate^11^ and SMRT-seq transcriptome data containing 89,477 unique sequences showed 59% complete Benchmarking Universal Single-Copy Orthologs (BUSCO) score^16^. These suggested that it is necessary to obtain more higher coverage transcriptome with certain data quality for solid molecular analysis of *C. morifolium*.

Full-length transcripts can greatly increase the accuracy of transcriptome characterization and are beneficial for subsequent functional studies. PacBio SMRT-seq has been deployed to investigate expressed gene isoforms in various organisms, including several plant species^18–25^. The long reads delivered by SMRT-seq report full-length transcripts sequenced from their 5′-ends to their polyadenylated tails for transcriptome reconstruction without a reference genome sequence and without assembling fragments to resolve complete isoform sequences. Therefore, SMRT-seq is suitable for transcriptome analysis of polyploid species with complex genome. However, SMRT-seq has a relatively high error rate (14%)^26^. Considering this, it is necessary to improve sequence quality through construction of consensus sequence reads. Very high coverage and a relatively high cost are necessary to achieve highly accurate whole transcriptomes using only SMRT-seq technology^27,28^. Therefore, a combination of SMRT technology and high-coverage short-read sequences enable the acquisition of accurate and nearly complete transcriptomes at moderate cost. This combination of transcriptome sequencing has been used to reveal the transcriptome of tetraploid *Solanum tuberosum*^29^, which has a genome size of 1.6–1.8 Gb^30^. In *S. tuberosum*, de novo assembled datasets using four different algorithms or settings and one PacBio single-molecule long-read isoform sequencing (Iso-seq) dataset were combined and clustered into one dataset having 90% BUSCO score^29^. The examples of the combination of the transcriptome from different approaches in the plants are limited. We used a combination of transcriptome sequencing methods in *C. morifolium* for the first time. Additionally, we incorporated the error-corrected long-read sequences to increase the transcriptome variation. To our knowledge, no studies are available concerning a combination of transcriptome sequencing for segmental hexaploid plants with nearly 10 Gb genome.

**Figure 1:**
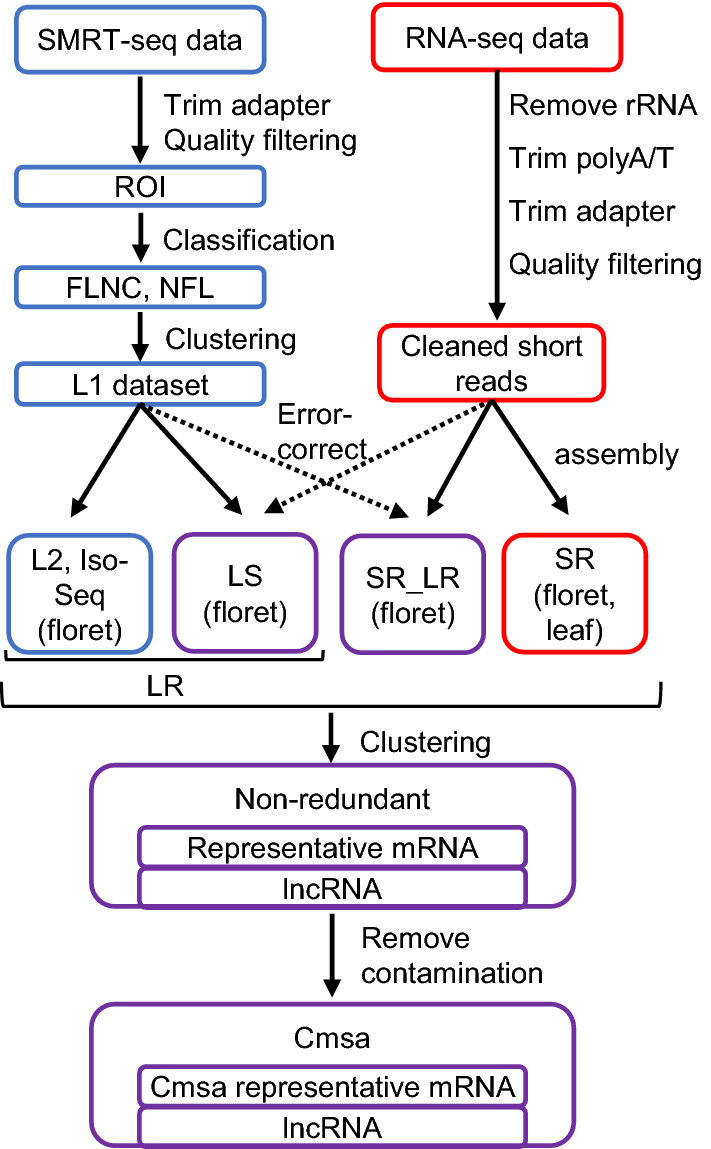
Flow of dataset preparation and analysis. SMRT-seq raw reads were processed into ROI, classified into FLNC and NFL sequences, and clustered into the L1 dataset. The L1 dataset was further quality filtered into the L2 dataset. The L1 dataset was also error-corrected with cleaned short reads to produce the LS dataset. RNA-seq data were cleaned and used for de novo assembly. The L2, LS, SR_LR, and SR datasets were combined and clustered into one non-redundant dataset. The contaminated sequences were removed from the non-redundant dataset. The final dataset was named the Cmsa dataset and deposited in the DDBJ database. ROI: reads of insert. FNLC: full-length non-chimeric. NFL: non-full length. L1: long-read 1. L2: long-read 2. LS: L1 sequences error-corrected with cleaned short-read sequences. LR: long-read-derived dataset including the L2 and LS datasets. SR: short-read, de novo assembled dataset. SR_LR: short-read, de novo assembled dataset with long-read assistance. Cmsa: *Chrysanthemum morifolium* ‘Sei Arabella’ dataset.

The main objective of this study was to obtain reliable high-coverage transcript sequences for molecular analysis in *C. morifolium.* We focused on anthocyanin biosynthesis pathway because accumulation of reddish anthocyanin pigments in ray-floret strongly influences its commercial value and is sensitive to even relatively high temperatures. In Japan, floral development of *C. morifolium* occurs at nighttime temperatures of 10–20 °C. During summer in temperate zones like Japan, it is difficult to commercially produce naturally red-colored *C. morifolium* cultivars, especially those with fine pink coloration, without heat-exchange equipment. Understanding the detailed molecular components that generate sensitive flower colors is the first important step to analyzing regulatory mechanism and will have important implications for the rational manipulation of flower color. For this purpose, we reproduced the color-dulling effect of temperature under artificial conditions. We applied a combination of transcriptomes from SMRT-seq technology and from Illumina RNA-seq technology (Fig. 1). To increase the transcriptome variation, we applied the error-correction step to long reads and recovered sequences eliminated during the Iso-seq pipeline. We also performed de novo assemblies with the assistance of long-read data to achieve broad transcript coverage. We confirmed the reliability of the obtained dataset via BUSCO analysis, mapping rate, homology searches. We showed that the combination of long- and short-read sequences with correction steps increased transcript coverage and quality compared with only using long-read- or short-read-derived transcripts. Using the combined dataset, we found transcripts encoding anthocyanin biosynthesis pathway genes, analyzed their expression with qRT-PCR and confirmed that our dataset useful for detail molecular analysis. The approach in this report provided the way to obtain reliable transcriptome data of *C. morifolium* and will enhance the functional analysis of important traits including ray-floret coloration.

## Results

### Preparation of dataset using SMRT-seq and RNA-seq

To obtain the comprehensive transcriptome dataset, we performed an analysis flow to completely use SMRT-seq and RNA-seq data (Fig. 1). The Iso-seq pipeline has several steps to polish sequences. First, raw reads were processed into error-corrected reads of insert (ROIs). Second, ROIs were classified into four categories, namely, full-length non-chimeric (FLNC), chimeric, non-full length (NFL), and short reads, by searching for the polyA tail signal and the 5′ and 3′ primers. Subsequently, the adapter sequences and polyA tails were removed. FLNC and NFL reads were polished and clustered into consensus sequences. Finally, the consensus sequences were filtered with the quality score and named as Iso-seq sequences. We selected the dataset before the final filtering step and named it as long-read 1 (L1) dataset and named the Iso-seq dataset long-read 2 (L2) dataset. We applied the error-correction step using the Hercules algorithm to the L1 dataset with RNA-seq data. We obtained de novo assembled sequences with or without assist of long-read sequences. Combination of these four kinds of datasets was intended to increase sequence variation. Then, we removed redundant sequences and clustered combined dataset. In the aspect of future molecular analysis, we focused on anthocyanin biosynthesis genes to verify the data quality.

**Figure 2:**
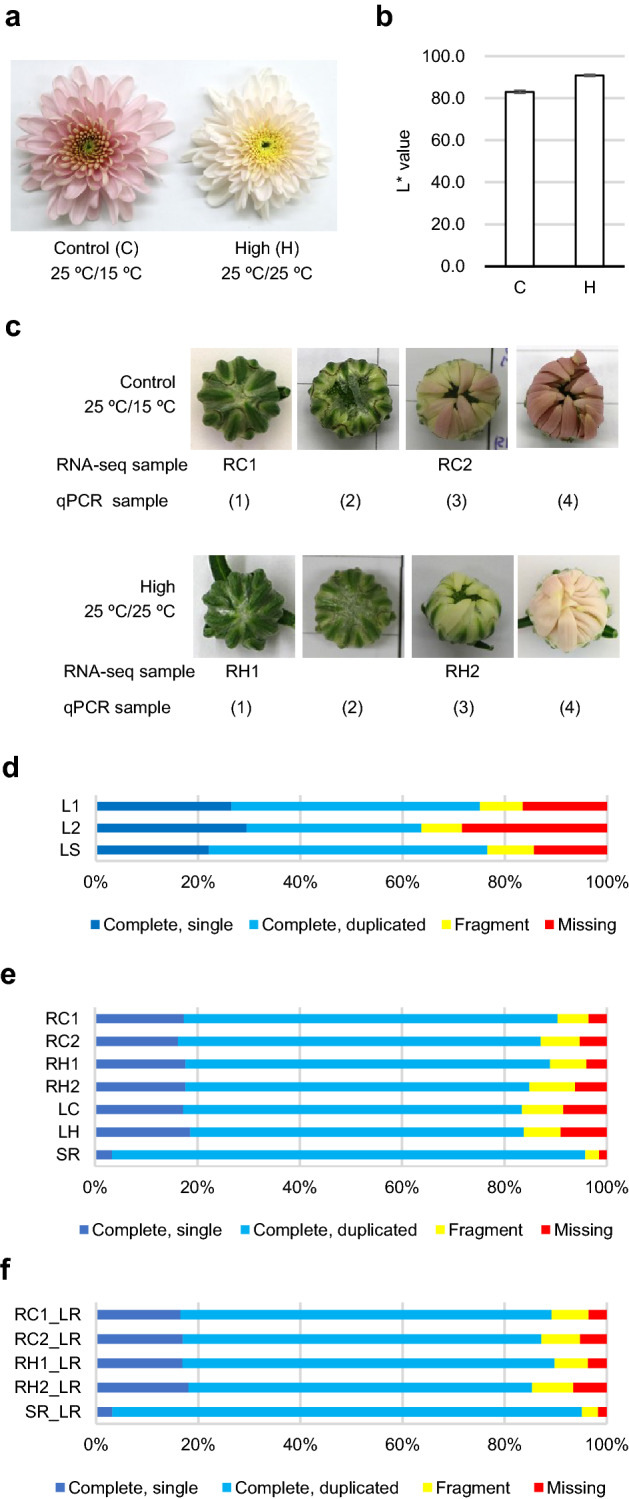
Samples used for SMRT-seq and RNA-seq and completeness of each dataset. (**a**) Photograph of ‘Sei Arabella’ flower grown under control (left, 25 °C/15 °C) or high nighttime temperature (right, 25 °C/25 °C) conditions. (**b**) L* value of a ‘Sei Arabella’ flower grown under control (C) or high (H) nighttime temperature conditions. Four biological replicates were used for the analysis, and error bars indicate standard deviation. L*: Perceptual lightness value of chromatic axes. (**c**) Buds and blooms, of which ray florets were used for analysis. For RNA-seq, RC1, RC2, RH1, and RH2 ray-floret samples were used. For qRT-PCR, ray florets from growth stages (1) to (4) were used. (**d**–**f**) Result of the BUSCO assessment of L1, L2 and LS dataset (**d**), and of RC1, RC2, RH1, RH2, LC, LH, and SR (**e**), and of RC1_LR, RC2_LR, RH1_LR, RH2_LR, and SR_LR (**f**) on embryophyta data. L1: long-read 1 dataset. L2: long-read 2 dataset. LS: long reads corrected with short reads. RC1: short-read sequences from ray florets under control conditions, stage 1. RC2: short-read sequences from ray florets under control conditions, stage 3. RH1: short-read sequences from ray florets grown at the high nighttime temperature, stage 1. RH2: short-read sequences from ray florets grown at the high nighttime temperature, stage 3. LC: short-read sequences from leaves grown under control conditions. LH: short-read sequences from leaves grown at the high nighttime temperature. SR: short-read-derived dataset including RC1, RC2, RH1, RH2, LC, and LH datasets. RC1_LR, RC2_LR, RH1_LR, and RH2_LR are short-read-derived dataset with long-read assistance and combined into SR_LR.

We cultured ‘Sei Arabella’, which have light pink-colored ray-florets. Nighttime temperature is an important factor in anthocyanin accumulation in *C. morifolium* cv. ‘Orchid Queen’ ray florets^31^. Cultivation at 25 °C leads to decreased anthocyanin accumulation in five cultivars^32^. Therefore, ‘Sei Arabella’ plants were cultured in a greenhouse until the appearance of the first flower buds and then transferred to growth chambers set at 25 °C/25 °C (high; H) or 25 °C/15 °C (control; C) (daytime/nighttime) at the same light intensity. We selected relatively moderate temperature to avoid an excess effect on ray-floret development. Under the high-temperature condition, the flowers were lighter in color than those of plants grown in the control condition (Fig. 2a). Perceptual lightness (L*) value of chromatic axes in the International Commission on Illumination color space also increased under the high-temperature condition (Fig. 2b). We extracted RNA from ray florets at different developmental stages and conditions, sequenced, and characterized the RNA using PacBio SMRT sequencing technologies. From SMRT-seq, 469,698 ROI sequences were obtained, classified into FLNC, chimeric, NFL, and short-reads categories (Table 1). The FLNC reads were polished using both FLNC and NFL reads and clustered into 180,137 transcript isoforms, which was called the L1 dataset, with a mean length of 1595 bp (Supplementary Fig. S1, Supplementary Table S1). Of the transcripts, 114,349 isoforms had high-quality scores (Quality > 0.99 in Quiver); they were corresponding to usual Iso-seq sequences and referred to as L2 transcript isoforms; the mean length was 1598 bp. The Iso-seq workflow discarded 36.5% of the L1 reads, likely caused by low sequencing accuracy.

**Table 1:** Summary of SMRT-seq.

Total reads	Total base (Gb)	Number of reads of inserts	Number of reads with 5′ sequence	Number of reads with 3′ sequence	Number of reads with poly-A sequence	Number of FLNC reads
481,325	14.91	469,698	380,772	388,249	376,578	323,101

To obtain deep transcriptome data reflecting the color-dulling phenomena, we selected before and after the initiation of petal colorization under control conditions and performed strand-specific RNA-seq of ray-florets at two developmental stages and two temperature conditions (Fig. 2c, Table 2). Ray-floret samples from the control condition (RC1, RC2) or from the high nighttime temperature condition (RH1, RH2) were used. Leaf samples from the control condition (LC) or from the high nighttime temperature condition (LH) were also used. All the raw data were filtered and quality-trimmed to obtain clean short reads.

**Table 2:** Summary of RNA-seq.

	LC	LH	RC1	RC2	RH1	RH2
Raw bases (Gb)	6.4	7.5	7.4	9.2	7.9	7.7
Number of total raw reads	50,012,494	42,395,168	49,510,188	61,332,634	52,470,178	51,328,650
Number of clean reads	48,151,440	40,825,460	47,708,062	59,218,490	50,453,538	49,387,462

### Error correction of the L1 dataset with RNA-seq data increased the completeness and mapping rate of the long-read-derived dataset

To increase the full-length transcript sequence variation, we performed error correction of the L1 dataset with clean short reads using the Hercules program^33^. We named the error-corrected dataset as the long reads corrected with short reads (LS) transcript dataset. The LS dataset contained the same number of transcript isoforms (180,137 sequences) of mean length 1582 bp as the L1 dataset. The length distribution in the LS dataset was similar to that in L1 dataset (Supplementary Fig. S1). Then we estimated the completeness of each transcript dataset using the Benchmarking Universal Single-Copy Orthologs (BUSCO) program with the Embryophyta subset^34,35^ (Fig. 2d). BUSCO checks for essential single-copy orthologs that should be present in whole transcriptome datasets. The complete BUSCO in each dataset was 75.1% in the L1 dataset, 63.7% in the L2 dataset, and 76.6% in the LS dataset. To measure the mapping rate, cleaned short reads were aligned to the L1, L2, and LS datasets using Bowtie2 alignment software^36^. The average alignment rate of all the clean short reads was 86.8% in the L1 dataset, 84.9% in the L2 dataset, and 87.3% in the LS dataset (Supplementary Fig. S1). These results suggested that unique but low-quality transcript isoforms were eliminated from the L1 dataset in the quality-filtering step and that error-correction step could be used to recover these sequences and increase the full-length transcript sequence variation.

### De novo assembled transcripts from strand-specific RNA-seq widely cover the transcriptome

We performed de novo assembly of clean short reads using the Trinity assembler^37^. The assembly was based on the RNA-seq library (RC1, RC2, RH1, RH2, LC, and LH), and each assembled sequence was combined into the short-read-derived (SR) dataset (Supplementary Table S1). The BUSCO score of each assembled sequence was 86.4% on average, and that of the combined SR dataset was 95.8% (Fig. 2e). Additionally, each ray-floret RNA-seq library was assembled using the L1 dataset. These assembled sequences were named RC1_LR, RC2_LR, RH1_LR, and RH2_LR respectively and combined into the short-read- and long-read-derived (SR_LR) datasets (Supplementary Table S1). The BUSCO score of each assembled sequence was 87.9% on average, and that of the SR_LR dataset was 95.1% (Fig. 2f). These results indicated that the combination of assembled datasets slightly increased the transcript variation.

**Figure 3:**
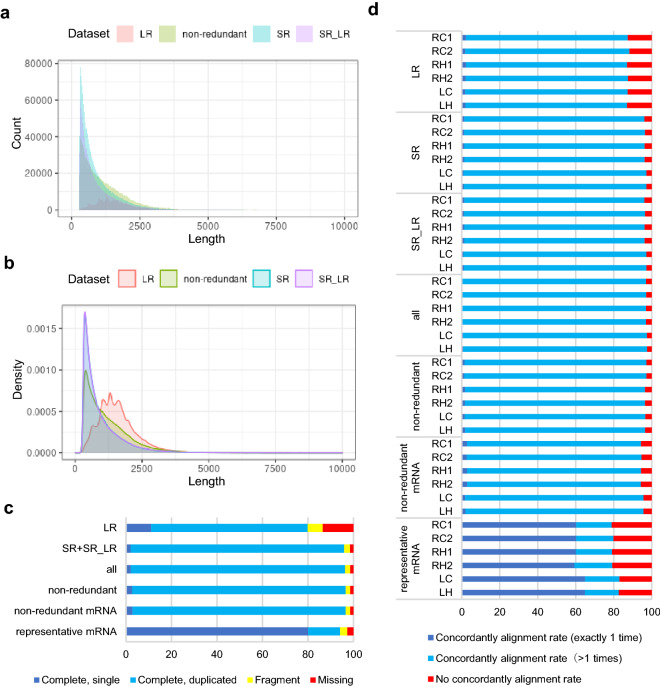
Comparison of long-read and short-read derived datasets and their combined non-redundant transcript dataset in the aspect of sequence length, completeness, and mapping rate. (**a**,**b**) Nucleotide lengths in each dataset was indicated using histogram (**a**) and density plot (**b**). (**c**) Result of the BUSCO assessment of each dataset on Embryophyta data. (**d**) Alignment rate of each short-read sequence toward the combined dataset using the Bowtie2 aligner. LR: long-read derived sequences including L1 and LS dataset. SR: short-read-derived dataset including RC1, RC2, RH1, RH2, LC, and LH datasets. SR_LR: short-read- and long-read-derived dataset including RC1_LR, RC2_LR, RH1_LR and RH2_LR. RC1: short-read sequences from ray florets under control conditions, stage 1. RC2: short-read sequences from ray florets under control conditions, stage 3. RH1: short-read sequences from ray florets grown at the high nighttime temperature, stage 1. RH2: short-read sequences from ray florets grown at the high nighttime temperature, stage 3. LC: short-read sequences from leaves grown under control conditions. LH: short-read sequences from leaves grown at the high nighttime temperature. The growth stages of the ray-floret samples are indicated in Fig. 2.

A feature of the de novo assembled sequences was an abundance of short sequences (< 1000 bp, Fig. 3a,b). To evaluate the L1 assistance on the production of the short-read-derived dataset, we combined the SR datasets from ray florets, namely RC1, RC2, RH1, and RH2 (SR_ray_floret) with the SR_LR dataset as SR_ray_floret + SR_LR. Both the BUSCO score (95.5%) and the Bowtie2 alignment rate (96.5% on average) of the SR_ray_floret + SR_LR dataset were slightly increased from either the SR_LR dataset (95.1% complete BUSCO, 96.0% Bowtie2 alignment rate on average) or SR_ray_floret dataset (95.2% complete BUSCO, 95.9% Bowtie2 alignment rate on average, Supplementary Fig. S2). This indicated that these two assembled datasets did not completely overlap and could increase the variety of transcript isoforms.

### Combining the four kinds of dataset into one dataset, filtering and classification of transcripts

To increase the transcript variation and coverage of transcriptomes, we combined all the transcript datasets (L2, LS, SR, and SR_LR). This all dataset had high BUSCO (95.4%) and Bowtie2 alignment rates (94.2% on average). To remove the fragments and redundant sequences, we filtered the redundant sequences and classified mRNA and long-non coding RNA (lncRNA) using the EvidentialGene package^38,39^. EvidentialGene used the predicted amino acid coding sequences to remove the redundant sequences, and to select non-redundant mRNA transcripts. The non-redundant mRNA transcripts contained 928,645 sequences. The non-redundant mRNAs were clustered by coding-region similarity, and classified as representative or variant transcripts by the length and completeness of the coding regions. The representative mRNA transcripts contained 92,854 sequences. LncRNAs were selected from the all transcript datasets excluding non-redundant mRNA transcripts and their similar transcripts. Then the remained transcripts with short length (≤ 300 bp) or redundant sequences were removed, and classified into lncRNA. These steps resulted in 276,519 lncRNA (Supplementary Table S1).

The 1,205,164 non-redundant transcript isoform dataset had improved BUSCO (96.6%, Fig. 3c) and Bowtie2 alignment rate (96.8% on average; Fig. 3d) compared with the LR, SR, and SR_LR datasets. The non-redundant mRNA dataset had same BUSCO score (96.6%) and a small decrease at Bowtie2 alignment rate (94.9% on average). This indicated that the lncRNA dataset constitutes part of transcriptome. The representative mRNAs remained high BUSCO score (94.1%; complete single 80.1% and complete duplicated 14.0%). This supported that representative mRNAs actually reflected majority of *C. morifolium* ray-floret transcriptome with little drain. The Bowtie2 alignment rate of the representative mRNAs were lower (80.5% on average) than the non-redundant dataset. This indicated that the other mRNA sequences were also constitute complex transcriptome and is necessary to understand *C. morifolium* ray-floret transcriptome.

### Assessing the quality and annotating representative mRNA

**Figure 4:**
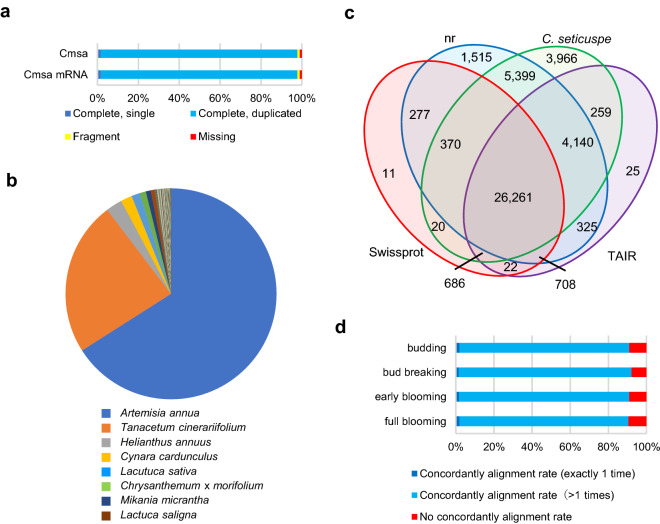
Features of the *Chrysanthemum morifolium* ‘Sei Arabella’ transcript dataset (Cmsa). (**a**) Result of the BUSCO assessment of the Cmsa and Cmsa mRNA dataset on Embryophyta data. (**b**) Similarities of representative mRNA transcript isoform sequences with those of other species were analyzed from the results of a blastp search against the NCBI nr database. (**c**) Venn diagram of homology search results of the amino acid sequences predicted from the representative mRNA sequences of the Cmsa dataset. The blue circle indicates nr (NCBI nr database). The green circle indicates *C. seticuspe* (CSE_r1.1.pep database). The red circle indicates Swiss-Prot (Uniprot Knowledgebase (UniProtKB/Swiss-Prot). The purple circle indicates TAIR (*Arabidopsis thaliana* protein sequences (Arabidopsis_thaliana.TAIR10.pep.all.fa)). (**d**) Alignment rate of short-read sequence from *C. morifolium* cultivar ‘Jinba’ pefal toward the Cmsa data using the Bowtie2 aligner.

To evaluate the dataset quality and predict the function of each sequence, homology searches were performed with the predicted amino acid sequences of the representative mRNAs (92,854 isoforms) against the NCBI NR database (Supplementary Table S2) and the Swiss-Prot database (Supplementary Table S3). We then removed sequences for which the best matches were sequences derived from non-vascular plant species as contaminated sequences. These contaminated sequences included sequences from spider mites or bacteria, which might live in plants. The 1,198,366 clean sequences formed the *Chrysanthemum morifolium* ‘Sei Arabella’ (Cmsa) transcript dataset including 921,854 mRNA and 276,512 lncRNA (Table 3). The BUSCO scores of the Cmsa and Cmsa mRNA datasets were both 97.9% with the Embryophyta subset (Fig. 4a). This Cmsa mRNA included 89,734 Cmsa representative mRNA.

**Table 3:** Summary of Cmsa dataset.

Dataset	Cmsa	Cmsa mRNA	Cmsa representative mRNA	LncRNA
Number of total sequences	1,198,366	921,854	89,734	276,512
Average length (bp)	1205	1332	972	783
Min. length (bp)	280	280	294	292
Max. length (bp)	18,923	18,923	18,923	16,067

We then analyzed sequences similar to the Cmsa representative mRNA sequences in the NR database (Fig. 4b). The top eight hit species were the Asteraceae species, including *Artemisia annua* (25,467 isoforms), *Tanacetum cinerariifolium* (9381 isoforms), *Helianthus annuus*, *Cynara cardunculus*, *Lactuca sativa*, *C. morifolium*, *Mikania micrantha*, and *Lactuca saligna*. These sequences occupy 96.7% of the NR hit isoforms, suggesting that these isoform sequences are highly probable. Homology searches using the predicted amino acid sequences of the Cmsa representative mRNAs (89,734 isoforms) were conducted (Table 4) with the *Arabidopsis thaliana* protein database (Ensemble, TAIR 10, PEP; Supplementary Table S4) and the *Chrysanthemum seticuspe* peptide database (CSEr1.1; Supplementary Table S5). From the Venn diagram of sequences showing homology in each database, we found 3966 sequences homologous only to *C. seticuspe* (Fig. 4c). This suggests that there are unique sequences to the chrysanthemum species. The remaining sequences, which were not homologous to the analyzed database, might contain unique sequences to *C. morifolium*.

**Table 4:** Summary of the homology search of amino acid sequences from Cmsa representative mRNAs.

	Total	nr	Swiss-Prot	TAIR	*C. seticuspe*	No annotation
**All**						
Sequence number	89,734	38,995	28,355	32,426	41,101	45,749
Ratio to total number	100%	43.46%	31.60%	36.14%	45.80%	50.98%
**≥ 100 aa**						
Sequence number	52,194	36,779	30,526	26,888	36,825	13,066
Ratio to total number	100%	70.47%	58.49%	51.52%	70.55%	25.03%

When we focused on long amino acid sequences from Cmsa representative mRNAs (≥ 100 aa, 52,194 isoforms), the ratio of sequences with homology to each database increased (Table 4). InterProScan was used to find protein motifs and Gene Ontology (GO) categories of representative mRNAs (Supplementary Tables S6–S9). There were 121,312 InterProScan descriptions. The most abundant InterProScan descriptions were the protein kinase-like domain superfamily (1669 isoforms) and leucine-rich repeat domain superfamily (1606 isoforms) (Supplementary Tables S6, S7). A total of 98,521 isoforms were classified into 27 cellular component categories, 457,344 isoforms were classified into 37 molecular function categories, and 531,485 isoforms were classified into 25 molecular function categories of GO (Supplementary Tables S8, S9). Transcription factors (TFs) were searched using the list of *Arabidopsis thaliana* TFs annotated in the PlantTF database and revealed 1428 putative TFs in the Cmsa representative mRNA dataset (Supplementary Table S4).

We further analyzed the Cmsa dataset that could be used as reference for the published NGS data from the *C. morifolium* cultivar, ‘Jinba’^40^. ‘Jinba’ has white ray-floret and is not considered to have a direct relationship to ‘Sei Arabella’. Illumina short read data of ‘Jinba’ petals were mapped onto the Cmsa dataset (Fig. 4d). Data from four developmental stages, budding, bud breaking, early blooming, and fully blooming, showed high mapping scores (90.95, 92.18, 90.92, and 90.56%, respectively). These suggested that the Cmsa dataset could be used as reference at least the petal sample from other *C. morifolium* cultivars.

### SMRT-seq increased the variation of sequences with complete open reading frame

To analyze the contribution of each sequencing method toward Cmsa mRNA dataset, we analyzed the dataset origin of the Cmsa representative mRNA sequence (Table 5). We found that 27.6% of sequences were fully derived from long-read sequences or with the assistance of long-read sequences. The mean length of the nucleotide sequences in the LR dataset was longer than that of the SR dataset. When we focused on the full-length open reading frame (ORF) sequences containing a start methionine and stop codon in the same translation flame, 61,116 sequences appeared to contain full-length ORFs. Among these, 32.5% of sequences were fully or partially derived from long-read sequences. These suggested that the SMRT-seq process increased the variation of longer mRNAs, especially those encoding complete ORFs.

**Table 5:** Summary of the sequence origin in the Cmsa representative mRNA dataset.

	Number of sequences	Ratio to total sequence number (%)	Mean length (bp)	Min. length (bp)	Max. length (bp)	Number of full-length sequences*	Ratio to total full-length sequences (%)
LR	9178	10.2	1649	307	18,923	8180	13.4
SR_LR	15,613	17.4	1138	301	13,166	11,668	19.1
SR	64,942	72.4	837	294	13,541	41,268	67.5

*Number of sequences predicted to have a start methionine and stop codon in the longest translation flame.

### Characterization of the anthocyanin biosynthesis pathway gene orthologs

To evaluate whether our dataset was useful for analyzing molecular function and gene expression, we searched the transcript isoforms of anthocyanin biosynthesis pathway genes by conducting a homology search with known anthocyanin biosynthesis pathway enzymes from the Kyoto Encyclopedia of Genes and Genomes (KEGG) website^41^ (Supplementary Table S10). The anthocyanin biosynthesis pathway (Supplementary Fig. S3) from chalcone synthase (CHS) to UDP-glucose:flavonoid 3-*O*-glucosyltransferase (3-GT) is conserved in most plant species. In *C. morifolium*, the cyanidin 3-*O*-(3″,6″-di-*O*-malonyl)glucoside and cyanidin 3-*O*-(6″-*O*-malonyl)glucoside are the major red pigments^42^, which are produced from cyanidin 3-*O*-glucoside via anthocyanin 3-*O*-glucoside-6″-*O*-malonyltransferase (3MaT1) and anthocyanidin 3-*O*-glucoside-3″,6″-*O*-dimalonyltransferase (3MaT2)^43^. In the 31 clusters encoding orthologs of anthocyanin biosynthesis pathway genes, 3MaT1, and 3MaT2, there were 875 isoforms. These sequences contained both transcripts encoding putative full-length and unique fragment sequences. We validated the homology search result with alignments of representative sequences and query sequences (Supplementary Figs. S4–S11).

### Expression analysis using qRT-PCR validate the Cmsa dataset reliability

**Figure 5:**
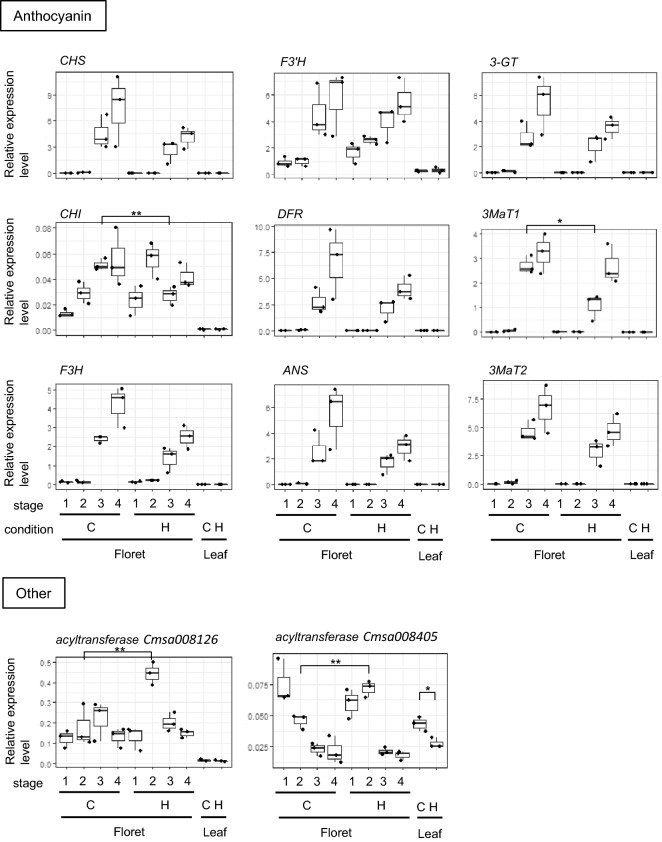
Changes in expression of anthocyanin biosynthesis gene orthologs as indicators of dataset quality. Changes in the expression of the anthocyanin biosynthesis gene orthologs determined by qRT-PCR. qRT-PCR was performed to evaluate the expression of *CHS*, *CHI*, *F3H*, *F3′H*, *DFR*, *ANS*, and *3-GT* orthologs, *3MaT1* and *3MaT2*. Two *acyltransferase* genes were analyzed as controls for other metabolite pathways. The vertical bar indicates the relative expression level using Cmsa000988 as reference. Each point indicated a relative expression level. The bold bar indicates the mean of three biological replicates. Student two-sample t-test was performed between control and high nighttime temperatures at the same growth stages. *p-value < 0.05. **p-value < 0.01. H: samples cultured at 25 °C/25 °C (high nighttime temperature). C: samples cultured at 25 °C/15 °C (control condition). The growth stages of the ray-floret samples are indicated in Fig. 2.

There were 572 anthocyanin biosynthesis gene orthologs estimated to be expressed in at least one RNA-seq library (Supplementary Table S11). We selected transcript isoforms that seemed to be regulated in a temperature-dependent and/or growth-dependent manner. We designed primers of the selected transcript and analyzed their expression using qRT-PCR (Fig. 5). Because of the high sequence similarity in each cluster, the qRT-PCR results might have included sets of highly similar transcript isoforms. All of the selected transcript isoforms expressed in floret samples. Among them, expression of *CHS* (*Cmsa011121*), *flavanone 3-hydroxylase* (*F3H*, *Cmsa006810*), *dihydroflavanol 4-reductase* (*DFR*, *Cmsa011753*), *anthocyanidin synthase* (*ANS*, *Cmsa011788*), *3-GT* (*Cmsa006941*), *3MaT1* (*Cmsa008409*), and *3MaT2* (*Cmsa008349*) transcript isoforms seemed to be affected by growth stage and temperature at night condition. The expression of these genes seemed to increase between stage 2 and stage 3 under control condition. When we compare two temperature conditions, high temperature at night condition seemed to repress the expression level of *CHS*, *F3H*, *DFR*, *ANS*, *3-GT*, *3MaT1*, and *3MaT2* transcript isoforms at stage 3 and/or at stage 4. Among them, *CHI* and *3MaT1* transcript isoforms at stage 3 showed a statistically significant difference between the two temperatures. The expression of *flavonoid 3*′*-hydroxylase* (*F3*′*H*, *Cmsa012340*), and *chalcone isomerase* (*CHI*, *Cmsa012818*) transcript isoforms was affected by the growth stage at control temperature, but their expression patterns were different from those of other anthocyanin biosynthesis genes. These expression pattern of anthocyanin biosynthesis gene orthologs were co-incident with the repression of ray-petal color change under control condition (Fig. 2) and with the TPM value from RNA-seq data (Supplementary Fig. S12). Two *acyltransferase* transcript isoforms (*Cmsa008126* and *Cmsa008405*) have highly similar sequences to *3MaT1* and *3MaT2* (Supplementary Fig. S11) but would function in another pathway and show dissimilar expression patterns to *3MaT1* and *3MaT2*. Additionally, carotenoid biosynthesis gene candidates showed low expression with no obvious difference in heat treatment at least with the TPM value from RNA-seq data (Supplementary Table S12). These data suggested that a series of anthocyanin biosynthesis gene orthologs increased their expression in accordance with ray floret development, and high night temperature partially repressed them. Therefore, transcripts of our dataset correctly reflected the ray-floret appearance of ‘Sei Arabella’ and would be useful for molecular analysis.

## Discussion

In this study, we performed large-scale sequencing of cDNA for transcriptome reconstruction of *C. morifolium* ray-floret. A combination of full-length cDNA sequencing with SMRT technology and Illumina stranded RNA-seq was used to obtain the *C. morifolium* transcriptome and to create a reference for transcript expression profiling. We initially obtained L2 sequences from the usual Iso-seq workflow. The Iso-seq workflow discarded 36.5% of the L1 dataset, likely caused by low sequencing accuracy. To increase the sequence variation, we applied a hybrid error-correction algorithm with Illumina short reads and recovered 65,788 sequences. The error-corrected LR sequences (LS dataset) increased both the BUSCO score (from 63.7 to 76.6%) and the Bowtie2 alignment rate (from 84.9 to 87.3%). Therefore, the hybrid error-correction was useful for expanding the full-length transcript dataset. On the other hand, both the BUSCO score and Bowtie2 alignment rate indicated that long-read derived sequences from 14 Gb raw data were not sufficient to cover whole ray-petal transcriptome of *C. morifolium*. Transcriptome reconstruction using short-read sequencing by de novo assembly is cost-effective and yields high coverage data; however, it is biased toward short transcript isoform lengths. It is difficult to reconstruct full-length transcripts from short-read sequences with high accuracy^44^. SMRT sequencing eliminates these drawbacks, delivering full-length transcripts. The combination of SMRT sequencing and short-read sequencing is highly beneficial for complete transcript sequencing at reduced cost. High-throughput sequencing technology has been used to obtain transcript datasets in *C. morifolium* (summary in Supplementary Table S13). Our analysis offers the most extensive list of non-redundant sequences in *C. morifolium*. This method will be one of the choices to analyze *C. morifolium* transcripts and also applicable to other plants with large complex genome.

In our study, we found 875 isoform sequences encoding anthocyanin biosynthesis gene orthologs from 921,854 mRNAs in the Cmsa mRNA dataset (Supplementary Table S10). Both the raw data and the Cmsa datasets are available at DDBJ (BioProject ID PRJDB10476). We demonstrated that the Cmsa dataset had high mapping scores (> 90%) with the different cultivar ‘Jinba’ petal RNA-seq data (Fig. 4d). We believe our dataset could sufficiently cover the *C. morifolium* ray-floret transcriptome, provide sequence information in reliable quality, and be useful for molecular analysis. The TPM value of the anthocyanin biosynthesis gene orthologs from 9 genes showed similar pattern to the corresponding qRT-PCR results (Fig. 5, Supplementary Fig. S12). This also supported that our Cmsa dataset is useful as the reference of RNA-seq analysis. The statistical analysis with repeated RNA-seq experiment will reveal other genes in same regulatory mechanism.

The expressions of *CHS*, *CHI*, *F3H*, *F3′H*, *DFR*, and *ANS* were shown to be affected by growth temperature using qRT-PCR in *C. morifolium* ‘Pelican’^45^. They fixed the temperature at 20 °C or 30 °C all the day and used ray floret samples at the petal extension stage, early vertical stage, and petal fully open stage; the former two stages were almost consistent with our stages 3 and 4. The tendency that the expression of anthocyanin biosynthesis genes were affected by growth temperature was same with our result. This supported that our data are reliable to analyze gene expression. The temperature of 25 °C at night inhibited the colorization (Fig. 2) but did not completely inhibit the expression of anthocyanin biosynthesis genes (Fig. 5). The continuous repression of the expression of anthocyanin biosynthesis genes would have led to visible differences in ray-floret color. We showed that the set of anthocyanin biosynthesis pathway genes (*CHS*, *F3H*, *DFR*, *ANS*, *3-GT*, *3MaT1*, and *3MaT2*) had similar expression patterns (Fig. 5). This suggested that the same regulatory mechanism affects the expression of these transcripts and is expected to cause color repression that occurs at high nighttime temperatures. On the contrary, expression of *F3*′*H* ortholog transcript was not restricted under high temperature at night conditions. Analyzing the regulatory mechanism of *F3*′*H* gene and applying that mechanism to other temperature-sensitive anthocyanin biosynthesis pathway genes would be interesting to control ray-floret colorization. It is well-known that transcriptional complex containing MYB, bHLH and WD40 regulates the orchestrated expression of anthocyanin biosynthesis genes with activators and repressors^46^. The analysis of regulatory mechanisms of these anthocyanin biosynthesis transcripts will be important to develop temperature-resistant flower color in *C. morifolium* using biotechnology. And our dataset will promote to investigate the regulatory mechanism of the anthocyanin biosynthesis.

We have previously demonstrated that the clustered regularly interspaced short palindromic repeats (CRISPR)-associated protein 9 (Cas9) system is applicable in *C. morifolium*^5^. Recently, genome editing using the transcription activator-like effector nucleases (TALEN) system could simultaneously confer male and female sterility in *C. morifolium*^47^. However, it still requires enormous effort and a lot of time to determine target sequences for genome editing in *C. morifolium*. This inhibits our further use of genome editing technology to modify agronomical traits in *C. morifolium*. Our dataset and/or methodology could be used to analyze the molecular regulatory mechanisms of important traits, including anthocyanin repression, affected by high nighttime temperature and to obtain the transcript sequences more easily. These improvements will facilitate the application of genome editing technology to *C. morifolium*.

In conclusion, we demonstrated that the combination of SMRT-seq data and Illumina short-read data created and refined a comprehensive ray-floret reference transcriptome in hexaploid *C. morifolium*. We obtained functionally annotated transcript isoforms, including anthocyanin biosynthesis gene orthologs. Our dataset offer fundamental information for the accurate analysis of the molecular function in ray-floret of *C. morifolium* and would facilitate the modification of *C. morifolium* traits using biotechnology. Our methodology to obtain transcriptome data would facilitate the molecular analysis of plants with large and complex genome including *C. morifolium*.

## Methods

### Plant materials, RNA preparation, and flower color measurement

The plant of *C. morifolium* ‘Sei Arabella’ cultivar (Inochio Seikoen; https://www.seikoen-kiku.co.jp/en/) was commercially available and provided from Inochio Seikoen Inc. with the permission to use the ‘Sei Arabella’ for research purposes. The experimental research on plants complies with the relevant institutional, national, and international guidelines and legislation. Plants were cultivated in a greenhouse under short-day conditions (8 h/16 h, day/night). After the first flower bud had emerged, the plants were transferred to growth chambers (LH-411SP, Nippon Medical and Chemical Instruments) set at 25 °C day/25 °C night (high; H) or 25 °C day/15 °C night (control; C) at the same light intensity. Fluorescent lights were set at a photosynthetic photon flux density of 108 μmol s^−1^ m^−2^ for the first and last hour of daylight, and of 382 μmol s^−1^ m^−2^ for the remaining 6 h.

For RNA extraction, samples were immediately frozen in liquid nitrogen, and stored at − 70 °C. Total RNA was extracted using TRIzol (Thermo Fisher Scientific), treated with DNase I (QIAGEN), and further purified using the RNeasy Mini Kit (QIAGEN).

For color measurement, the outermost ray petal florets in full bloom was evaluated by a CD100 spectrum colorimeter (Yokogawa Test and Measurement). The L* value for lightness of the CIE L*a*b* color space was measured at least three times from four biological replicates.

### PacBio long-read library preparation, sequencing, and data processing

Ray-floret samples were collected from the outermost and second-outermost layers at four stages: (1) bud was 10 mm in diameter and completely covered with involucral bracts, (2) ray florets were just emerging from the bud, (3) the edges of the ray florets started to become pink under control condition, and (4) the tips of ray florets began to open (Fig. 2). The ray florets of small buds (< 7 mm) grown under control condition and just bloomed ray florets under both temperature conditions were also used. The total RNA samples from each ray floret were pooled. Library preparation, sequencing, and read processing were performed at the Beijing Genomics Institute. RNA integrity was determined using the Bioanalyzer 2100 (Agilent Technologies, RIN > 10, 28S/18S ratio > 1.7). One microgram of total RNA was used for cDNA synthesis using the Clontech SMARTer PCR cDNA Synthesis Kit (Takara Bio). KAPA HiFi DNA Polymerase (Roche) were used for amplification of the cDNA. The cDNA libraries were size-fractionated (< 5 kb) and sequenced using two SMRT cells with v2.1 chemistry on a PacBio Sequel instrument (Pacific Biosciences). Raw reads were processed using the Iso-seq pipeline using SMRT Link v2.3.0 into error-corrected ROI. ROI were classified into four categories as FLNC, chimeric, NFL, and short reads. FLNC and NFL. Reads were polished and clustered into consensus sequences using Quiver in SMRT Link.

### Illumina short-read library preparation, sequencing, and data processing

RNA samples of ray florets at stage 1 and 3 and of fully developed leaf with five compound leaves were used. Library preparation, sequencing of stranded RNA-seq (2 × 150 bp paired-end), and raw data filtering were performed at Novogene. The mRNA was enriched using oligo(dT) beads and randomly fragmented. The cDNA was synthesized with random hexamers and DNA polymerase I. The double-stranded cDNA library was prepared through PCR enrichment and sequenced on a Novaseq 6000 (Illumina). Then, filtering process was performed: removal of reads containing adapters, removal of reads containing N > 10% (N represents undetermined bases), and removal of reads with > 50% low-quality bases (Qscore ≤ 5).

Read processing of Illumina short reads was performed at Genebay Inc. Reads representing ribosomal RNA were removed using the SortMeRNA v3.0.3^48^. PolyA/T sequences and adapters and 3′-end low-quality sequences trimmed using fqtrim (v0.9.7; https://ccb.jhu.edu/software/fqtrim/) and cutadapt ver.2.5^49^. We further cleaned the short-read data with fastp v0.21.0^50^ to remove reads with average quality scores < 30.

### Computational error correction and assemble of transcripts

The long-read data were error-corrected with the clean short-read data from ray-floret samples using the Hercules program^33^.

The clean short reads were used for de novo assembly using the Trinity assembler v2.11.0^37,51^. The settings were the following: --seqType fq, --SS_lib_type RF, --min_contig_length 300, and --trimmomatic. For a combined assembly using both short reads and long read, the following settings were used: --seqType fq, --SS_lib_type RF, --min_contig_length 300, --trimmomatic, and --long_reads.

### Quality assessment of transcript dataset

Transcript datasets were mapped onto BUSCO embryophyte_odb10 subsets (eukaryota, 2020-08-05) using BUSCO v 4.1.3 and v 5.2.1^34,35^ for the final Cmsa dataset. The following settings were used: -m transcriptome and -l embryophyta_odb10. The concordant alignment rate of filtered short reads for each transcript was calculated using the Bowtie2 aligner (v 2.3.5.1)^36^ with the options --no-unal and -k 20.

The ‘Jinba’ transcriptome data^40^ were obtained from the NCBI SRA database (SRR3921639, SRR3921658, SRR3921660, and SRR3921662). The downloaded fastq files were interleaved with cutadapt and mapped onto the Cmsa dataset using the Bowtie2 aligner (v 2.3.4.1) with the options -no-unal and -k 20.

### Decreasing redundancy, classification, and clustering by deduced amino acid length of transcripts

Combined datasets were filtered and clustered with the tr2aacds pipeline (tr2aacds4.pl) of the EvidentialGene package (evigene20may20)^39^. There were five steps: (1) amino acid coding sequences are computed, and the longest coding region of each sequence is used for subsequent analyses; (2) coding regions in nucleotide sequences are compared using fastanrdb software of exonerate-2.4.0^52^; (3) redundant fragments are removed using CD-HIT-EST (cd-hit-v4.8.1-2019-0228)^53^; (4) the remaining transcripts are grouped by coding-region similarity using blastn (NCBI blast-2.6.0+)^54^; and (5) classified by the length and completeness of the coding regions as representative mRNA sequences and other variants. The representative sequences were all numbered as CmsaXXXXXXXt1, where X indicated the cluster number.

The long non-coding RNAs (lncRNAs) were classified using the tr2ncrna pipeline (v 2020.02.25) in EvidentialGene. This consists of two steps: (1) remove the non-redundant mRNA, their redundant sequences, sequences similar to non-redundant mRNAs, short sequences (≤ 300 bp); (2) group transcripts by similarity using megablast.

### Homology search of transcript datasets

The NCBI NR protein database was downloaded on 2020-10-08. Swiss-Prot protein sequences were downloaded from the Uniprot Knowledgebase (UniProtKB/Swiss-Prot) on 2021-01-19. Homology searches using blastp (NCBI blast-2.10.1+) were performed with the following parameters: -outfmt 6 -max_target_seqs 1 or 5 -evalue 1e-5. Gene names were assigned to the highest score hits. Sequences with homology to non-vascular plant species or non-plant organisms against the NCBI NR database, or with homology to non-plant species in the Swiss-Prot database were removed as contamination. From the non-redundant mRNA dataset, sequences belonging to the same cluster of contamination were removed. The vector and adapter sequences, which had homology to the Univec database (bitscores > 50), were trimmed and remained dataset was named as Cmsa transcript dataset.

Protein sequences of *Arabidopsis thaliana* (Arabidopsis_thaliana.TAIR10.pep.all.fa) and *C. seticuspe* (CSE_r1.1.pep) were downloaded from Ensembl Plants (release 49) and from Mum GARDEN (http://mum-garden.kazusa.or.jp/), respectively. Homology searches for the Cmsa representative amino acid sequences were performed using blastp (NCBI blast-2.10.1+ except CSE r1.1.pep for blast-2.11.0+) with the same parameters indicated above. To predict TF, the *A. thaliana* TF list (Ath_TF_list.txt) from PlantTFDB (http://planttfdb.gao-lab.org/download.php) were to the blastp result against *A. thaliana* protein sequences.

A functional domain search by InterProScan (v. 5.36–75.0) was performed with the default parameters. GO information assigned to InterPro domains was extracted from the results, and GOslim analysis was conducted using Agbase^55^ to categorize GO terms into three categories: Biological Process, Cellular Component, and Molecular Function.

Carotenoid biosynthesis gene search was performed using blastp with evalue cutoff 1E-50 using *A. thaliana* and tomato query sequences in KEGG website. The IDs of the query sequences are indicated in Table S12.

### Transcript abundance prediction

To predict transcript abundance, transcripts per million (TPM) was calculated using the RSEM tool^56^, and Bowtie2^36^ was used for the alignments. The non-redundant dataset was used for TPM calculation. Clean short reads of each RNA-seq library were used for estimating expression levels.

### Prediction of anthocyanin biosynthesis gene orthologs

Sequences from the pathway map of the KEGG website were used as query of anthocyanin biosynthesis gene (Supplementary Table S10). The transcripts of anthocyanin pathway gene orthologs were searched using blastp with the deduced amino acid sequences of non-redundant mRNA isoforms with the following parameters: -max_target_seqs 500 -evalue 1e−50. For *F3′H* ortholog, the -max_target_seqs parameter was set to 2000. If all isoforms belonging to a cluster had low bitscores, the cluster was removed. The minimum bitscores were the following: 300 for *F3H*, *DFR*, *CHS*, *3MaT1*, *3MaT2*, *ANS*, and *3GT* and 400 for *F3′H*. The remaining isoforms and query sequences were aligned using the MAFFT program (v7.450) in Geneious prime software (Biomatters) with following parameters: algorithm, auto; scoring matrix, 200PAM/k = 2; gap open penalty, 1.53; offset value, 0.123; and automatic determination of sequence direction. Based on the alignment, we manually selected sequences and used them as queries for an NCBI blast search against the NR database to confirm that the best hit result was annotated as the expected function. The functional domains were searched using InterProScan (Supplementary Figs. S4–S11).

### Reverse transcription and qRT-PCR

RNA samples of ray florets at stage 1, 2, 3 and 4, and of developed leaf with five compound leaves were used. The ReverTra Ace-α-cDNA synthesis kit with oligo (dT) was used to synthesize cDNA from 500 ng of total RNA (Toyobo). qRT-PCR was performed using the SYBR Premix Ex TaqII (Tli Rnase H Plus) kit (Takara Bio) on a Thermal Cycler Dice Real Time System II (Takara Bio). The threshold cycle (C_t_) was determined by the second derivative maximum method. Statistical analysis of qRT-PCR data was performed using R (version 4.1.2). The relative quantitation method was used to compare transcript expression levels. The reference transcript was selected as a constitutively expressed transcript using a TPM value with the following criteria: (1) the expected count is above 100, and (2) the TPM ratio between each sample was between 0.8 and 1.25. The list of primer sequences are in Supplementary Table S14.

## Supplementary Information

Supplementary Figures.

Supplementary Tables.

## Data Availability

The raw sequencing data are available in the DDBJ Sequence Read Archive database at https://www.ddbj.nig.ac.jp/dra/index.html and can be accessed with accession numbers DRA012773 and the BioSample accession SAMD00244182-SAMD00244188 (SSUB015942). The assembled sequence data are available in the DDBJ Transcriptome Shotgun Assembly division at https://ddbj.nig.ac.jp/public/ddbj_database/tsa/TSA_ORGANISM_LIST.html and can be accessed with ICRW010000001-ICRW011198366. Both the raw and assembled data can be accessed with BioProject ID PRJDB10476.
