# Supplementary material for: Combination of long-read and short-read sequencing provides comprehensive transcriptome and new insight for Chrysanthemum morifolium ray-floret colorization
Source: Sci Rep. 2022 Oct 25;12:17874. doi: 10.1038/s41598-022-22589-z (PMC9596691; doi:10.1038/s41598-022-22589-z)
Supplement: Supplementary file 1 — Supplementary Figures. [file 41598_2022_22589_MOESM1_ESM.pdf]

Title: Combination of long-read and short-read sequencing provides comprehensive transcriptome and new insight for *Chrysanthemum morifolium* ray-floret colorization

Scientific Reports

Mitsuko Kishi-Kaboshi[1, 3, *], Tsuyoshi Tanaka[2], Katsutomo Sasaki[1], Naonobu Noda[1], Ryutaro Aida[1]

[1] Institute of Vegetable and Floriculture Science, National Agriculture and Food Research Organization (NARO), Fujimoto 2-1, Tsukuba, Ibaraki, 305-0852, Japan

[2] Research Center for Advanced Analysis, National Agriculture and Food Research Organization (NARO), Kannondai 2-1-2, Tsukuba, Ibaraki 305-8518, Japan

Corresponding Author:

Mitsuko Kishi-Kaboshi

E-mail address: mkaboshi@affrc.go.jp

a

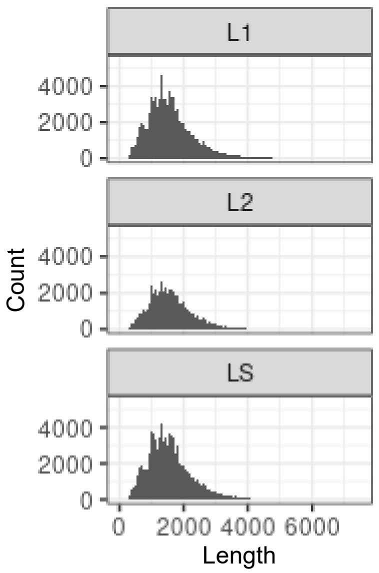

b

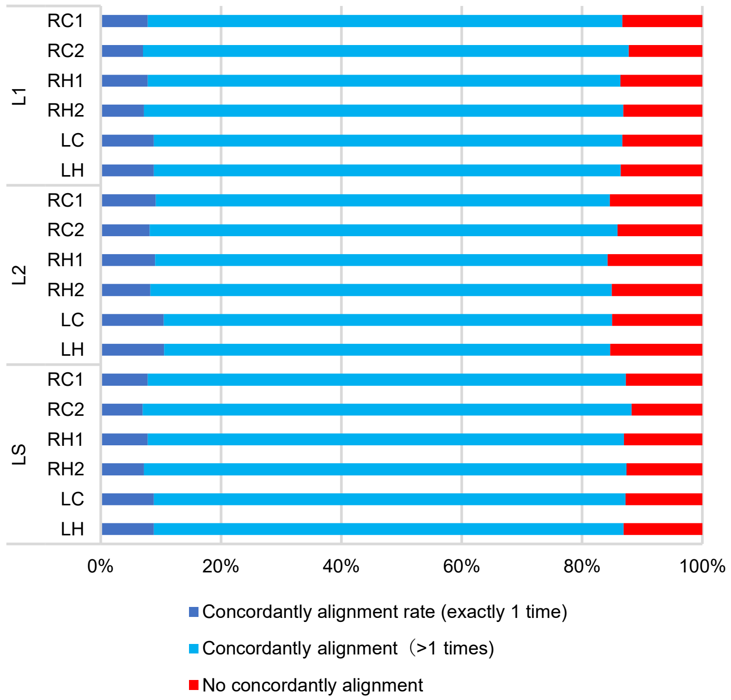

Figure S1. Features of long-read derived datasets.

SMRT-seq data were processed into L1 dataset, then high quality sequences were selected as L2 dataset. The L1 dataset was corrected using short-read sequence and named as LS dataset.

a. Density plot of nucleotide lengths in each dataset.

b. Mapping rate of each short-read sequences toward long-read derived datasets using the Bowtie2 aligner.

L1: long-read 1. L2: long-read 2. LS: L1 sequences error-corrected with cleaned short-read sequences. RC1: short-read sequences from ray floret at control condition stage 1. RC2: short-read sequences from ray floret at control condition stage 3. RH1: short-read sequences from ray floret at high temperature at night condition stage 1. RH2: short-read sequences from ray floret at high temperature at night condition stage 3. LC: short-read sequences from leaf at control condition. LH: short-read sequences from leaf at high temperature at night. Stages of ray floret samples were indicated in Fig. 2.

a

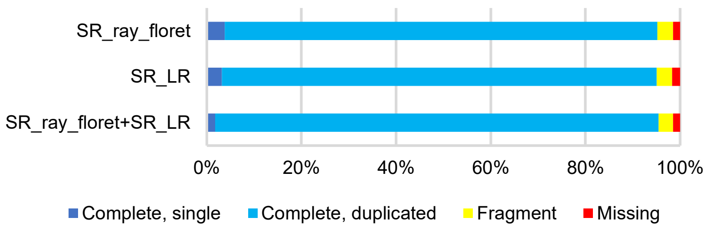

b

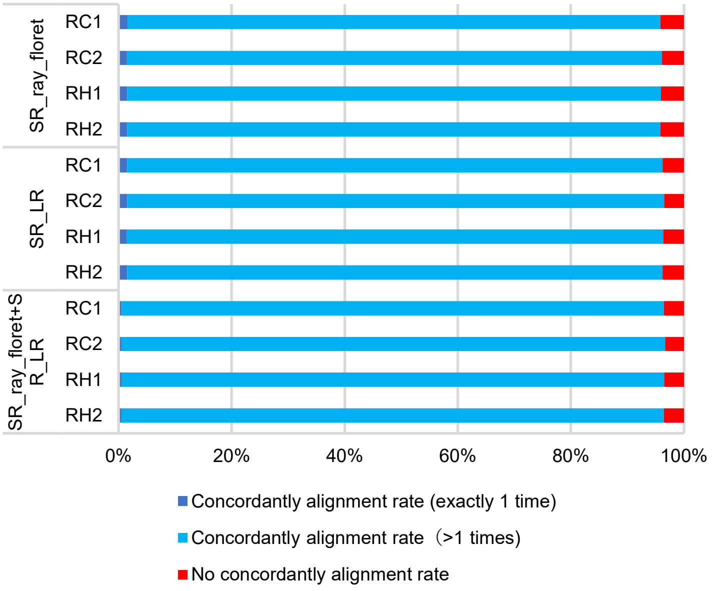

Figure S2. Comparison of *de novo* assembled datasets from ray floret with or without long read assistance.

a. Result of the BUSCO assessment of each dataset on embryophyta data.

b. Mapping rate of each short-read sequences toward long-read derived datasets using the Bowtie2 aligner.

SR_ray_floret: de novo assembled dataset from RC1, RC2, RH1, and RH2. SR_LR: de novo assembled dataset from RC1, RC2, RH1, and RH2 with assistance of long-read sequences. RC1: short-read sequences from ray floret at control condition stage 1. RC2: short-read sequences from ray floret at control condition stage 2. RH1: short-read sequences from ray floret at high temperature at night condition stage 1. RH2: short-read sequences from ray floret at high temperature at night condition stage 2. Stages of ray floret samples were indicated in Fig. 2.

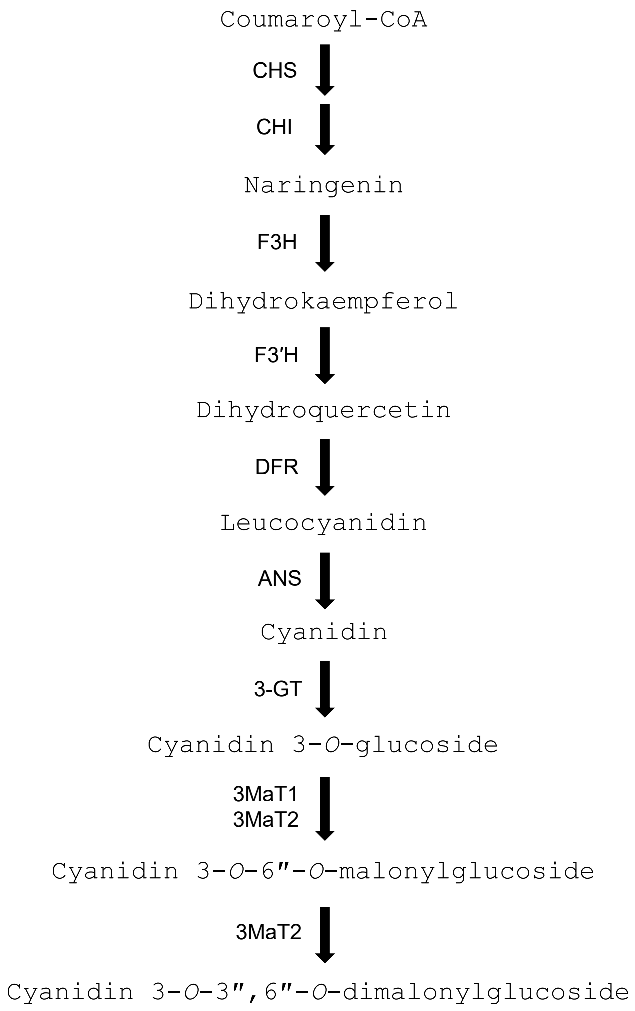

Figure S3. Flow of anthocyanin biosynthesis.

Black solid arrows indicate the enzymatic reactions.

```
Consensus        MXXMAXXXXXXXIRXAQRAXGPATILAIGTATPXNCVXQAXYPDYYFRITKSEHMXDLKEKFXRMCDKSMIRKRYMHLTE   80
Cmsa011121t1     ------MVSIQEL.K....E.............P...L.SE.............RK......T........N....Y...   74
CHS_AT5G13930    -MV..GASSLDE..Q....D...G.......N.E.H.L..E.........N....T......K......T....H.....   79
Cmsa010624t1     .ASSPAVVDVDA..K....Q.............S...Y..D..............T......Q..............I..   80
Cmsa009913t1     ---..SLTDIAA..E....Q.............A...Y..D..............V......K.................   77
Cmsa010627t1     ---..SLTDIAA..E....Q.............A...Y..D..............V......K.................   77

Consensus        EXLKENPNXCXYMAPSLDXRQDXVVVEVPKLGKEAATKAIKEWGQPKSKITHLXFCTTSGVDMPGADYQLTKLLGLRPSV  160
Cmsa011121t1     .I...K..L.A.......D...I..............R...............V...............I..........  154
CHS_AT5G13930    .F.....HM.A.......T...I.............V...............VV..........................  159
Cmsa010624t1     .Y......M.E.......A...L......................F........V..........................  160
Cmsa009913t1     .Y......L.E.......A...V...............................I..........................  157
Cmsa010627t1     .Y......L.E.......A...V...............................I..........................  157

Consensus        KRFMMYQQGCFAGGTVLRXAKDLAENNKGARVLVVCSEITAVTFRGPXDTHLDSLVGQALFGDGAAAXIVGSDPDXXVGE  240
Cmsa011121t1     ..................M.............M..............D...................I......IVD.-.  233
CHS_AT5G13930    ..L...............I........R...................S.............S.....L.......TS...  239
Cmsa010624t1     ..................L............................N...................V.......LS.-.  239
Cmsa009913t1     ..................L.........D..................N...................V.......LTK-.  236
Cmsa010627t1     ..................L............................N...................V.......LS.-.  236

Consensus        XPJFEMXSAAQTILPDSEGAIDGHLREVGLTFHLLKDVPGLISKNIEKXLXZAFXPLGIXDWNSJFWIAHPGGPAILDQV  320
Cmsa011121t1     K.I...A..S.................A................H...S.IE..Q....D....L...............  313
CHS_AT5G13930    K.I...V..........D............................V.S.DE..K....S....L...............  319
Cmsa010624t1     R.L...V.........................................A.TQ..S....N....I...............  319
Cmsa009913t1     R.L...I.........................................A.TQS.S....S....I...............  316
Cmsa010627t1     R.L...V.........................................A.TQ..S....N....I...............  316

Consensus        EXKLGLKEEKMRATRHVLSEYGNMSSACVLFIJDEMRXKSAXXGXXTTGEGLXWGVLFGFGPGLTVETVVLHSXXXXJXX  400
Cmsa011121t1     .E..A.TPD.L.....................LN...HS..TD.FN......E....................VSI----  393
CHS_AT5G13930    .I..............................L....R...KD.VA......E....................VPL----  399
Cmsa010624t1     .L.........K...Q................L....K..IED.KT......D....................LPTTISM  399
Cmsa009913t1     .L..............................I....K...EE.AA......D....................LPTTISV  396
Cmsa010627t1     .L..............................I....K...EE.AA......D...................TASQPLYR  396

Consensus        XXDYG                                                                             405
Cmsa011121t1     -----                                                                             389
CHS_AT5G13930    -----                                                                             395
Cmsa010624t1     AS---                                                                             401
Cmsa009913t1     A----                                                                             397
Cmsa010627t1     LH...                                                                             401
```

Figure S4. Alignment of deduced amino acid sequences of *CHS* orthologs.

The Cmsa046550 cluster has only fragment sequence and was eliminated from alignment. Black box indicates chalcone/stilbene_synt_N domain (IPR001099). Blue box indicates chalcone/stilbene_synt_C domain (IPR012328). Red box indicates active site of chalcone/stilbene synthase (IPR018088). Only representative sequences are shown.

```
Consensus       MAXPXSXTSJXVEXIVFPXSVKPPGXTXTLFLGGAGVRGMEIQGNFVKFTGIGVYLEDKAIPXLAXKWKGKTAXELXBSV   80
CFI_SAUME       ..P.P.S..IQ..S....P......A.T..................................S..V.......A..TD..   80
Cmsa012818t1    ..T.S.A..LN..N....S......D.N..................................L..G.......E..VN..   80

Consensus       ZFXRDIVTGPFXKFXQVTMILPLTGKQYSEKVSEMCXGVWKAXGTYTDADXATIXKFLEVFKDENFXPGXSILFTTSPXG  160
CFI_SAUME       Q.Y........E..S.....................I.....Q.......T...E...........L..S........T.  160
Cmsa012818t1    E.F........K..T.....................V.....H.......G...D...........P..A........D.  160

Consensus       SLTISFSKDGXIPEAAXXVLENXKLAQXVIESVIGXXGVSPXXKQSLASRLXDXMXXFDEKATXBXEXXJXXNGL  235
CFI_SAUME       ..........N.....TV....R....T.......EH....EA........S.F.TQ......ANV.SQIGL---  232
Cmsa012818t1    ..........M.....NI....E....A.......KN....AT........A.L.NH......TDA.PNLSK...  235
```

Figure S5. Alignment of deduced amino acid sequences of *CHI* orthologs.

CFI_SAUME: AAM48130.1 chalcone isomerase [Saussurea medusa]. Black box indicates chalcone isomerase domain (IPR016087). Only representative sequences are shown.

```
Consensus        MAPXXLTXXXXXSXXXXXFVRDEDERPKVXYNXFSBEIPVISLXGIDDVXESSGGIKXXRXEICXXIXXACEBWGIFQVV   80
F3H_At3g51240    ...GT..ELAGE.KLNSK...........A..V..D.......A.....D-------GK.G...RQ.VE...N.......   73
Cmsa012340t1     ...IS.-KWGDN.LHENR...........P..K..N.......K.....E.......SR.A...EK.IK...D.......   79

Consensus        DHGVDTXXXXXMTRLARXFFXXPXEXKLRFDMXGGKKGGFIVSSHLQGEAVQDWREIVTYFSYPXXXRDYSRWPDKPXXW  160
F3H_At3g51240    ......NLVAD......D..AL.P.D......S...............................VRN..........EG.  153
Cmsa012340t1     ......KMLSE......E..EM.A.E......T...............................IKA..........KE.  159

Consensus        XXVTEXYSEXLMXLXCKLLEVLSEAMGLEKEXLXBACVDMDQKXVVNYYPKCPQPDLTLGLKRHTDPGTITLLLQDQVGG  240
F3H_At3g51240    VK...E...R..S.A................S.TN........I....................................  233
Cmsa012340t1     RA...K...D..G.G................A.KD........V....................................  239

Consensus        LQATRDXGKTWITVZPXEGAFVVNLGDHGHXLSNGRFKNADHQAVVNSNXSRLSIATFQNPAPBAXVYPLKVXEGEKXIX  320
F3H_At3g51240    ......N.......Q.V.............F..................S.............D.T......R....A.L  313
Cmsa012340t1     ......G.......E.I.............Y..................T.............N.I......N....S.M  319

Consensus        EEXITFXXMYKXKMXXDLELARLKKLAKXXXXXXEXXKPXXXIXA                                    365
F3H_At3g51240    ..P...AE...R..GR............EERDHK.VD..VDQ.F.                                    358
Cmsa012340t1     ..A...MD...K..ST............AKQQDL.KV..IES.L.                                    364
```

Figure S6. Alignment of deduced amino acid sequences of *F3H* orthologs.

Black box indicates DIOX_N domain (IPR026992). Blue box indicates Oxoglu/Fe_dep_dioxygenase (IPR005123). Only representative sequences are shown.

```
Consensus         MXILXXXXYXXIXXXXLXXXXXXFXXXRXXXXLPPGPXPWPIXGNLXXLXXXXHXXXAXXAXXYGPLMXLXXGXXDVVVX  80
F3PH_PETHY        .E..SLIL.TV.FSFL.QFILRS.FRK.YPLP.....K....I...VH.GPKP.QST.AM.QT.....Y.KM.FV....A  80
Cmsa006676t1      .N..TLLL.TF.TGLV.FYVSFN-KRN.RPGR.....T....V...PN.SSAM.HSL.SL.KK.....Y.RL.FL....V  79
Cmsa006810t1      .T..AFVF.AL.LGSV.-YVFLN-LSS.KSAR.....T....V...PH.GPIP.HAL.AL.KK.....H.RL.CV....A  78

Consensus         XSXXVAXQFLKXXDXNFXSRPPNSGXXXXXYNYQDXVFAPYGPRWRXLRKICSVHLFSXKXLDDFRHVRQXEVXXLTRXL  160
F3PH_PETHY        A.AS..A....TH.A..S.......AEHMA.....L.........M...........T.A.........D..KT...A.  160
Cmsa006676t1      S.SY..E....VN.G..A.......GKYIG.....M.........M...........P.T.........E..RI...I.  159
Cmsa006810t1      A.AS..A....VH.A..A.......AKHVA.....L.........L...........A.A.........E..AV...V.  158

Consensus         XXAXXXPVXLGXLLXXCXXNXLXXXMLGXRVFXBXSXXXXXXXXXEFKXMVVEXMVXAGXXNJXDFIPXLXXXDJQGXXXK  240
F3PH_PETHY        AS.GQK..K..Q..NV.TT.A.ARV...K...ADG.GDVDPQAA...S....M..V..VF.IG....Q.NWL.I..VAA.  240
Cmsa006676t1      VD.KNS..K..K..SM.TA.T.TQM...R...NNE.EGSEQTVD...D....L..L..EI.IS....P.ECL.L..LIK.  239
Cmsa006810t1      LS.GNS..Q..Q..NV.AT.A.ARV...R...GD---GIDRSAN...D....L..L..EF.LG....V.DLF.L..ITK.  235

Consensus         MKKLHXRFDXFLXXIXEEHKTXXXXXFGXMXDLLXTLISLKNDDADXXGGKLTDXEIKALLLNLFXXGTDXSSXTVEWAJ  320
F3PH_PETHY        .....A...A..TD.L....---GKI..E.K...S..........ND......T..........VA...T..S.....I  317
Cmsa006676t1      .....D...L..NK.I.....GKDNV..H-T...T......-....GE......I..........VG...S..L.....L  317
Cmsa006810t1      .....V...S..SK.V.....APGGL-.H-T...S......-....IE......T..........AA...T..S.....I  312

Consensus         AELIXXPXJLXQAXXEIDXVVGXDRLVXELDXXQLTXLZAXVKEXFRLHPXXXLSLPRIXSXSCEXBGYXIPKGSTLLXN  400
F3PH_PETHY        ....RN.KI.A..QQ...K...R....G...LA...Y.E.I...T.....STP......A.E...IN..F........L.  397
Cmsa006676t1      ....SH.KL.K..RD...N...R....T...VS...F.Q.V...T.....PAA......A.D...VN..Y........V.  397
Cmsa006810t1      ....RH.QI.K..RE...A...Q....T...LS...Y.Q.L...V.....STP......S.E...VD..Y........V.  392

Consensus         VWAIARDPXXWXDPLXFXPXRFLPGGEKPXXDVXXNDFEVXPFGAGRRICXGMXLGJRMVQLXXATLXXXFBWXLXXGXX  480
F3PH_PETHY        ........NA.A...E.R.E.........KV..RG.....I.........A..N..I.....MI...IHA.N.D.VS.QL  477
Cmsa006676t1      ........KV.T...K.Q.S.........GV..KV.....M.........V..S..L.....LV...VQA.D.E.TN.IE  477
Cmsa006810t1      ........KM.A...E.R.S.........GA..RG.....I.........A..S..L.....LI...VQT.D.E.AN.LE  472

Consensus         PEXLNMXEXXGLXXQRAXPLXVHPXPRLXXXXYXXX                                            516
F3PH_PETHY        ..M...E.AY..TL...D..V...R...EAQA.IG-                                            512
Cmsa006676t1      ..K...D.FF..NV...E..M...R...TPHV.RSA                                            513
Cmsa006810t1      ..M...E.AY..TL...A..M...K...APHV.ESI                                            508
```

Figure S7. Alignment of deduced amino acid sequences of *F3'H* orthologs.

F3PH_PETHY: Flavonoid 3'-monooxygenase of *Petunia x hybrida* (Cytochrome P450 75B2, Q9SBQ9.1). Black box indicates Cyt_P450_E_grp-I family (IPR002401). Red box indicates Cyt_P450_CS conserved site (IPR017972). Only representative sequences are shown.

```
Consensus          MKEDSPAIVCVTGASGFIGSWLVMRLLERGYIVRATTVRDPGDMKKVKHLLELPKAETNLTLWKADLALEGSFDEAIEGC   80
DFR_At5g42800      -MVSQKET.....................F...-......NL...Q...D..N.K.L........SE...Y.D..N..   78
CmDFR-OB_QPC96993  ..........................I........-............................................   79
Cmsa011753t1       ................................................................................   80
CmDFR-RM_QPC96994  ...................................-............................................   79

Consensus          HGVFHVATPMDFESKDPENEIIKPTIXGVLSIIRSCVKAKTVKKLVFTSSAGTVNVQKQQVPVYDESHWSDLDFIYSKKM  160
DFR_At5g42800      D...................V...VN.M.G.MKA........RRF...........EEH.KN....ND....E..M....  158
CmDFR-OB_QPC96993  ..........................E.....................................................  159
Cmsa011753t1       ..........................D............T........................................  160
CmDFR-RM_QPC96994  ..........................D.....................................................  159

Consensus          TAWMYFVSKTLAEKAAWKATKENNIDFISIIPTLVVGPFISPSFPPSLMTALSLITGXESHYSIIKQCQYVHLDDLCESH  240
DFR_At5g42800      .G...............DFAE.KGL...............TT.M....I....P..RN.A.....R.G.........NA.  238
CmDFR-OB_QPC96993  .........................................................V......................  239
Cmsa011753t1       .......................................................I.A......................  240
CmDFR-RM_QPC96994  .........................................................A......................  239

Consensus          IYLYEQPKAEGRYICSSHDATIHQLAKMIKEKWPEYQVPXKFEGIDDEIPXVSFSSKKLTDMGFKFKYDLEEMFRGAIKS  320
DFR_At5g42800      .F....AA.K............LTIS.FLRP.Y...N..ST...V.ENLKSIE...........N...S.....IES.ET  318
CmDFR-OB_QPC96993  .....N.................................T..........R.............................  319
Cmsa011753t1       .......................................A..........I.............................  320
CmDFR-RM_QPC96994  .......................................A..........I.............................  319

Consensus          CKEKGLLPYSTNXTKEGLVSSLVTKADTHEEEKLQXXXXXXXGXXPXXXXXXXXTXERTDAPMLAQQMCA            390
DFR_At5g42800      .RQ..F..V.LSYQSISEIKTKNENI.VK------TGDGLTD.MK.CNKTETGI.G..............            382
CmDFR-OB_QPC96993  ............D....................-------------------------------------            352
Cmsa011753t1       ............E......................IIHKVAT.NP.FSSSSKEH.W--------------            376
CmDFR-RM_QPC96994  ............E......................IIHKVAT.NP.FSSSSKER.W--------------            375
```

Figure S8. Alignment of deduced amino acid sequences of DFR orthologs.

CmDFR-OB: QPC96993.1 dihydroflavonol 4-reductase [Chrysanthemum x morifolium]. CmDFR-RM: QPC96994.1 dihydroflavonol 4-reductase [Chrysanthemum x morifolium]. CmDFR-OB lacks DFR function (Lim et al. 2020). Black box indicates Epimerase-deHydtase domain (IPR001509). Blue box indicates PANTHER entry region, DIHYDROFLAVONOL 4-REDUCTASE (PTHR10366:SF613). Only representative sequences are shown.

```
Consensus         MVXXXXXRVEXLAXSGIXXIXKEYIRXXXELXXIXBXFXEEXXZXXPQVPXXDLXXIXSXDXKXRXXCXXEJXKAXXXWG  80
ANS_At4G22880     --MVAVE...S..K...IS.P.....PKE..ES.NDV.L..KKEDG....TI..KN.E.D.E.I.EN.IE.LK..SLD..  78
Cmsa011788t1      ..IPINT...T..T...HQ.L.....TQD..TT.TNI.D..KKEQG....NI..ND.N.S.L.T.KK.CN.LV..ATE..  80
EpANS_BCM95298    ..ISANT...S..K...HE.P.....TQE..TT.TDI.H..NNQQC....TV..NH.N.D.P.A.EK.RH.IV..ATE..  80

Consensus         VMHJXNHGIXXDLXXRVKXAGEXFFXXXVXEKEKYXNDXXXGXXQGYGSXLANNAXGQLEWEDYFFHLXXPEEKRDLXXW  160
ANS_At4G22880     ...LI....PA..ME...K...E..SLS.E.....A..QAT.KI.....K.....S............AY.......SI.  158
Cmsa011788t1      ...IV....SG..IN...D...R..DQP.V.....S..IAS.KI.....K.....C............VF.......TV.  160
EpANS_BCM95298    ...LV....SN..IN...A...G..DQP.E.....S..IDS.NR.....R.....C............VF.......TI.  160

Consensus         PXXPXDYIXXTXEYAXXLRXLXTKXXXXXLSXGLGLXXXRLEKEVGGXEELJLQXKINYYPKCPQPELALGVEAHTDVSAL  240
ANS_At4G22880     .KT.S...EA.S...KC..L.A..VFKA..V....EPD........L...L..M..........................  238
Cmsa011788t1      .SK.S...PA.A...RQ..A.T..ILSV..L....EEG........L...I..F..........................  240
EpANS_BCM95298    .ST.T...PV.T...RQ..A.T..ILAA..I....DEG........M...L..L..........................  240

Consensus         TFXLHNMVPGLQLFYXGXWVTAKCVPDSIXMHIGDTLEILSNGKYKSILHRGLVNKEKVRISWAVFCEPPKXXKIXLKPLX  320
ANS_At4G22880     ..I............E.K..........V.......................................D..V....P  318
Cmsa011788t1      ..I............D.K..........I.......................................E..I....P  320
EpANS_BCM95298    ..K............N.Q..........I.......................................D..I....Q  320

Consensus         EXVSXEXPXXFPPRTFXXHXEHKLFXKXXXXXXXXXXX                                            358
ANS_At4G22880     .M..V.S.AK......AQ.I.....G.EQEELVSEKND                                            356
Cmsa011788t1      .T..E.N.PL......HQ.M.....K.KDDLVDPK---                                            355
EpANS_BCM95298    .T..K.E.PL......HH.M.....R.NNDKLNRNDTG                                            358
```

Figure S9. Alignment of deduced amino acid sequences of *ANS* orthologs.

EpANS: BCM95298.1 anthocyanidin synthase [Echinacea purpurea]. Black box indicates DIOX_N domain (IPR026992). Blue box indicates Oxoglu/Fe_dep_dioxygenase (IPR005123). Only representative sequences are shown.

```
Consensus             MEIXXXXXXXXXXXHVAVXAFPFXXHXXPLLXLXXXLAXXXPXXXXFSFFXTXXSNXXJFXXXXXXXXXXNIXXYBVXDG  80
UGT78K6               ---------MKNKQ...IFP...GS.LP...N.VLK..HIA.N-TS...IG.HS..AFL.TKRH---IPN..RVFTIS..  67
Cmsa006941t1          ...TGTINHGVREK....F....AS.PSL..T.ARK..SAA.N-VV....N.ET..KAL.SDQL---VYE..LP.N.W..  76
Cm3GT_QDY98361        ...TGTINHGVREK....F....AS.PSL..T.ARK..SAA.N-VV....N.ET..KAL.SDQL---VYE..LP.N.W..  76
3GT_VvGT1_100233099   ------MSQTTTNP....L....ST.AA...AVVRR..AAA.H-AV....S.SQ..ASI.HDSM-HTMQC..KS.D.S..  72
UGT78D2               ---MTKPSDPTRDS....L....GT.AA...TVTRR..SAS.S-TV....N.AQ..SSL.SSGDEADRPA..RV.DIA..  76
Pf3GT                 ---------MGFEL.IG.L....GT.AP...A.VRR..ASS.G-TL...LNSAE..AAL.NERT----YD..RAFD.W..  66
UFOG_GENTR            ---------MSPVS....L....GT.AA...T.VNR..ASA.D-II....S.SS.ITTI.SPTNLISIGS..KP.A.W..  70
Ph3GT                 --------MTTSQL.I.LL....GS.AA...T.VQK.SPFL.SDTI....N.SQ..TSI.SEG---SKPD..KV.N.W..  69
In3GT_BAR88263        --------MGSSEQ....L....AT.AA...S.VEQ.SAAF.SA-R....NNHD..SGL.RGRN--PAAGKVKA.D.W..  69

Consensus             XXXXXXFXGXPXEXXXLFJXXXPXXFXXXXXXAXXXXGXXXXCJXXDAFXWFXXDXAAXXXXXPWXXFWTAXXXSLSXHX  160
UGT78K6               IPEGHVPANN.I.KLD..LSTG.DNLRKGIEL.VAETKQSVT.IIA...VTSSLLV.-QTLNV..IA..PNVSC...LYF  146
Cmsa006941t1          IPKDYV.Q.K.L.EIN..LAEAEEE.RRVLKV.EVDI.LKVS.LVV...L..SG.V.-DEMNI..VA....GAS...A.F  155
Cm3GT_QDY98361        IPKDYV.Q.K.V.EIN..LAEAEEE.RRVLKV.EVDI.LKVS.LVV...L..SG.V.-DEMNI..VA....GAS...A.F  155
3GT_VvGT1_100233099   VAEGYV.A.R.Q.DIE..MRAA.ES.RQGMVM.VAET.RPVS.LVA...I..AA.M.-AEMGVA.LP....GPN...T.V  151
UGT78D2               VPEGYV.S.R.Q.AIE..LQAA.EN.RREIAK.ETEV.TEVK.LMT...F..AA.M.-TEINAS.IA....GAN...A.L  155
Pf3GT                 TPEGRI.T.THF.AVG..LKAS.GN.DKVIEE.EPKT.LKIC.LIT...L..AC.M.-QKRGL..VP....ASC...S.L  145
UFOG_GENTR            SPEGFV.S.N.R.PIEY.LNAA.DN.DKAMKK.VEDT.VNIS.LLT...L..AA.FS-EKIGV..IPV...ASC..CL.V  149
Ph3GT                 VTETNGNKPVGL.AIK..IQAT.TN.EKVMKE.EEET.VKFS.IFS...L..SYKL.-EKINV..IA....ASG...V.L  148
In3GT_BAR88263        TVAGEA-----LVTHEE.IMAM.GNYVKAIAE.EAET.TKFG.FLT...L..GG.L..ERGGV..IAL...GAC.I.A.L  144

Consensus             YTDXIRXXXXXXXXXXXXXXXZXXXXIPGFXSXXXXXDXPXXXXDXXBXXXXFXXXLXXMXXXLPXAXAVXXNSFZXLDP  240
UGT78K6               NI.L..DKCS------KDAKNATLDFL..-L.KLRVE.V.QDML.VGEKETL.SRT.NSLGVV..Q.K..VV.F.AE...  219
Cmsa006941t1          ...L..EKTAELK-GSVRPEDEIADL...-L.KVRLG.L.SGVV-FGNIESP.STM.HK.GRA..R.T..PL...QD...  232
Cm3GT_QDY98361        ...L..EKTAELK-GSVRPEDEIADL...-L.KVRLG.L.SGVV-FGNIESP.STM.HK.GRA..R.T..PL...QD...  232
3GT_VvGT1_100233099   ...E..EKIG-VS-GIQGREDELLNF...-M.KVRFR.LQEGIV-FGNLNSL.SRM.HR.GQV..K.T..FI...EE..D  227
UGT78D2               ...L..ETIG-VK-EVGERMEETIGV.S.-MEKIRVK.T.EGVV-FGNLDSV.SKM.HQ.GLA..R.T..FI...ED...  231
Pf3GT                 ...Q.VKAG-------TANQEQNLSF...-LEMATLT.L.PEVF-LDNSPSPLAITINK.VEK..KST..VL...EEI..  216
UFOG_GENTR            ...E..SRFAEFDI--AEKAEKTIDF...-L.AISFS.L.EELI-MEDSQSI.ALT.HN.GLK.HK.T..AV...EEI..  225
Ph3GT                 ...F..SNDETSL------------N....S.TLKIS.M.PEVM-AENLDLPMPSM.YN.ALN.HK.A..VL...EE...  215
In3GT_BAR88263        ...FV.SLAAATPTANGNGLDQKLKV...-M.ELSIGEM.GEIL-AKDLQAP.PGMIYN.ALK..G.N..VL...QN.E.  222

Consensus             XJTXXLXXXXXXXXLNIGPXXLXXXXXXXXXXXXXDXXXCJXWLXXXXXAXXSXXYJSFGTXXXPPPXEJXAXAEALEXX  320
UGT78K6               PLFVKYMRSKLQSL.YVV.LPCPQ-----LLLPEI.SNG.LS..DSKS--SR.VA.VC...VVS...Q.VV.V.....ES  292
Cmsa006941t1          DL.KN.SSK-LKNF.....FN.MS--KQTQSLKSD.EFS.IS..ENKK--PR.IA.I....TFK...H.IVEL.....ET  307
Cm3GT_QDY98361        DL.KS.SSK-LKNF.....FN.MS--KQTQSLKSD.EFS.IS..ENKK--PR.IA.I....TFK...H.ILEL.....ET  307
3GT_VvGT1_100233099   SL.ND.KSK-LKTY.....FN.IT--P--PPVI-PNTTG.LQ..KERK--PT.VV.I....VTT...A.LV.L.....AS  299
UGT78D2               TL.NN.RSR-FKRY.....LG.LS--STLQQLV-Q.PHG.LA.MEKRS--SG.VA.I....VMT...G.LA.I..G..SS  305
Pf3GT                 II.DD.KTK-FKNF..V..SI.ASPP----QATPD.ETG.LS..ADQTS-PK.VV.I....VIT..EN.LA.L.D...IC  290
UFOG_GENTR            II.NH.RSTNQLNI.....LQT--LS----SSIPPEDNE.LK..QTQK--ES.VV.L....VIN...N.MA.L.ST..SR  297
Ph3GT                 TINKD.KVK-LQKV.....LV.QPTSPK-KVLDAC.ERG.II..EKQK--EE.VV.L....VTTL..N.IV.V.....AK  291
In3GT_BAR88263        TV.DD.RSK-LQKVF....MI.RQAAATPKPPIIS.DHN.LP..DSLPP.SPPAV.L...SGLT...D.IV.L.....AK  301

Consensus             XXPFXWSLXXXXXXXXLPXGFLXRTXANXXGKXVXWAPQXZVLXHXXXGVFVTHCGWNSXLESXXXGVPXICRPFXGDQXJ  400
UGT78K6               GF..V.A.KESLLSI..K..VE..S--TR..V.S.V..SH..S.GSV........A..VM..VSN...M.....F...GI  370
Cmsa006941t1          KT..L..ISKDSEKHF.Q...E.IS..GT..V.P....VQ..E.FAI......G..S.V...IGV...M.....V...QI  387
Cm3GT_QDY98361        KT..L..ISKDSEKHF.Q...E.IS..GT..V.P....VQ..E.FAI......G..S.V...IGV...M.....LV..QI  387
3GT_VvGT1_100233099   RV..I...RDKARVH..E...EK.R--GY.MV.P....AE..A.EAV.A.........LW..VAG...L.....Y...RL  377
UGT78D2               KV..V...KEKSLVQ..K...D..R--EQ.IV.P....VEL.K.EAT...........V...VSG...M.....F...RL  383
Pf3GT                 RF..L...KDYAVKS..D...D..K--GF..I.A....QQ..A.RNV...........I...ISSC..L.....F...KL  368
UFOG_GENTR            KI..L...RDEARKH..EN.ID..S--TF..I.S....LH..ENPAI...........T...IFCR..V.G...F...KV  375
Ph3GT                 KF..I...KDNGIKN..T...E..G--QF..I.S....LEI.N.SAV...........I..GISC...M.....F...KL  369
In3GT_BAR88263        RA..L...KPHGVKH..E...E..K--EF..I.P....VQ..S.PGV.A.........T..AISF..CL.....Y...QI  379

Consensus             NXRXXEXXWXIGVXXEGGXFTKXXXXXXLXXXLXXXXLGXXXXXNXXXLKXXAXXAVXPXGSSXXNFXXLXXXXXXXXX  480
UGT78K6               AA.VIQDI.EV..IV..KV...NGFVKS.NLI.VQED-.KKIRD.ALKV.QIVQD..G.H.QAAED.NT.VEVISSS---  446
Cmsa006941t1          .TWMI.SV.E...RI...T...HGTRCA.EQV.SGDG.SERLKEKIEA..NL.HK.AE.N...NQ..KT.VDVVTGATL-  466
Cm3GT_QDY98361        .TWMI.SV.E...RI...T...HGTRCA.EQV.SGDG.SERLKEKIET..NL.HK.AE.N...NQ..KT.VDVVTGATL-  466
3GT_VvGT1_100233099   .G.MV.DALE...RI...V...ESGLMSCFDQI.SQEK-.KKLRE.LGA.RET.DR..G.K...TE..KT.VDLVSKPKDV  456
UGT78D2               .G.AV.VV.E..MTIIN.V...DGFEKC.DKV.VQDD-.KKMKC.AKK..EL.YE..SSK.R.SE..RG.LDAVVNII--  460
Pf3GT                 .S.MVQDS.K...RV...V...NEAVES.KKLMATEA-.MKIRE.VSL.REK.TA..K.E...SQ..KK.LEIIGAAESS  447
UFOG_GENTR            .A.MV.DV.K...GVK..V...EDETTRV.ELV.FSDK-.KEMRQ.VGR..EK.KD..KAN...TR..ES.LAAFNKLDS-  453
Ph3GT                 .S.MV.SV.Q..LQI...S...IGTISA.DTFFSEEK-.KVLRE.VKG..ER.LE..K.D...SK..KD.VELVKCHKLT  448
In3GT_BAR88263        .S.FV.SV.E...KV...K...DETLKA.NVV.DSDR-.KLLKE.VVK..GE.ME..K.N...TKD.QE.VHLLNGYF--  456
```

Figure S10. Alignment of deduced amino acid sequences of *3-GT* candidates and similar enzymes.

VvGT1 is a cyanidin 3-*O*-glycosyltransferase involved in the formation of anthocyanins. *UGT78K6* is an anthocyanidin 3-*O*-glucosyltransferase (GenBank accession no. AB185904) in butterfly pea (*Clitoria ternatea*). UGT78D2 is an anthocyanidin 3-*O*-glucosyltransferase in *Arabidopsis thaliana* (At5g17050). Pf3GT (BAA19659.1) is a flavonoid 3-*O*-glucosyltransferase in *Perilla frutescens*. UFOG_GENTR is an anthocyanidin 3-*O*-glucosyltransferase in *Gentiana triflora* (Q96493.1). Ph3GT is an anthocyanidin 3-*O*-glucosyltransferase in *Petunia x hybrida* (BAA89008.1). Black box indicates UDP_glucos_trans family (IPR002213). Red box indicates UDP_glycos_trans_CS domain (IPR035595). Blue dots indicates conserved residues in a wide range of glycosyltransferases (Brugliera et al. 1994).

a

```
Consensus          MASNSIVXILEQSRISSPPGTIGERSLPLTFFDIGWVPFPPVHHVFFYRFPHSKSHFLETVVPNLKXSLSLALQHFFPFA  80
Cmsa008409t1       .......K..........................................................N.............  80
3MaT1_Cm_AAQ63615  .......T..........................................................H.............  80

Consensus          SNLXVSPNXDDFGVIRKPEIRHVEGDYVALTFAECSTLDFNDLTGNHPRXCENFXPLVPPLGBXVKMXDXVTIPLFSVQV 160
Cmsa008409t1       ...S....V........................................E....H.......DL...S.Y.......... 160
3MaT1_Cm_AAQ63615  ...Y....A...........................-............K....Y.......NV...A.C.......... 159

Consensus          TYFXDSGISIGMTNHHXXXDASTRLGFLKXWTSIAKSGGDQSLXMNGSXPVLDRLIDXPKLDEYRLRHTSLETXYQPXSL 240
Cmsa008409t1       ...K............TVA..........A.............V....P........I...............L...W.. 240
3MaT1_Cm_AAQ63615  ...R............SLG..........V.............L....L........V...............F...P.. 239

Consensus          VGPTKKVRATFILSRTNINQLKKRVLTQIPTLEYISSFTVTCGYIWSCIAKSLVKMGEKKGEDELEQFICTADCRSRMDP 320
Cmsa008409t1       ................................................................................ 320
3MaT1_Cm_AAQ63615  ................................................................................ 319

Consensus          PIPSTYXGNCGAPCXTTIKNVVLSSENGFVFAAKLIGEAINKMVKNKEGILKDAERWHXXFKIPARKIGVAGTPKLNFYD 400
Cmsa008409t1       ......L.......I...........................................EG.................... 400
3MaT1_Cm_AAQ63615  ......F.......V...........................................DA.................... 399

Consensus          IDFGWGKPQKNETISIDYNGSVAINASKESTQDFEIGLCFSNMQMEAFADIFNHGLESEI                     460
Cmsa008409t1       ............................................................                     460
3MaT1_Cm_AAQ63615  ............................................................                     459
```

b

```
Consensus          MASNPIVTILEHSRISSPPGTIGERSLPLTFFDITWLAFPPVHHVFFYAFXHSKSHFLETVVPNLKHSLSLTLQHFFPFA  80
3MaT2_Cm_AAQ63616  ..................................................Q.............................  80
Cmsa008349t1       ..................................................P.............................  80

Consensus          GNLFVFPNADDXGVIRKPEIRHVEGDYVALTFAECXTLDFNDLTGNHPRECENFHPLVPPLGDLVKMXDYVTIPLLSVQV 160
3MaT2_Cm_AAQ63616  ...........Y.......................P...............................Y............ 160
Cmsa008349t1       ...........F.......................S...............................S............ 160

Consensus          TYFKDSGISIGMTNHHTVADASTRLGFLKAWTSIAKSGXDQSFVMNGSPXVLDRLIDXPKLDEFRLRHTSLETLYQPXSL 240
3MaT2_Cm_AAQ63616  ......................................R..........P.......I...................R.. 240
Cmsa008349t1       ......................................G..........L.......V...................W.. 240

Consensus          VGPTKKVRATFILSRTNINQLKKRVLTQIPTLEYISSFTVTCGYIWSCIAKSLVKMGEKKGEDELEQFIISVDCRSRMNP 320
3MaT2_Cm_AAQ63616  ................................................................................ 320
Cmsa008349t1       ................................................................................ 320

Consensus          PIPSTYXGNCGAPCXTTIKNVVLSSENGFVXXAKLISEAINKMVKNKEGILKDAERWHEGFKIPARKIGVAGTPKLNFYD 400
3MaT2_Cm_AAQ63616  ......L.......I...............FT................................................ 400
Cmsa008349t1       ......F.......V...............CA................................................ 400

Consensus          IDFGWGKPQKXEXISIDYXGSXAINASKESTQDFEIGLCFPNMQMKAFADIFNHGXESEX                     460
3MaT2_Cm_AAQ63616  ..........Y.A.....K..V.................................F...V                     460
Cmsa008349t1       ..........N.T.....N..I.................................L...I                     460
```

c

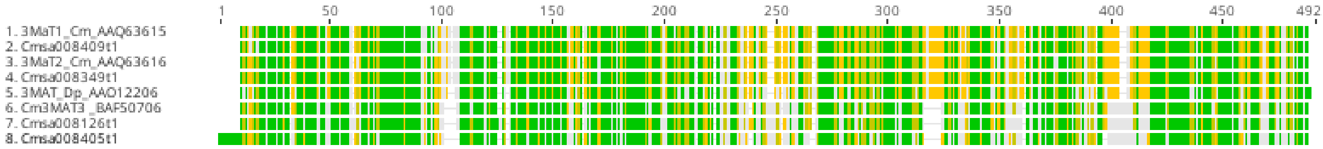

Figure S11. Alignment of deduced amino acid sequences of *3MaT1, 3MaT2*, and their homologs.

a. 3MaT1 (accession no. AAQ63615) and Cmsa008409t1 have 94.6% amino acid identity. The Cmsa180410 cluster has only fragment sequence and is eliminated from alignment.

b. 3MaT2 (accession no. AAQ63616) and Cmsa008349t1 have 96.1% amino acid identity. Only representative sequence is shown.

c. Sequences from 3MaT1, 3MaT2 and their homologs are aligned. 3MaT3 might be an acyltransferase for phenolics other than anthocyanins (Unno et al. 2007). 3MaT3 (accession no. BAF50706) and Cmsa 008126t1 have 90.5% amino acid identity.

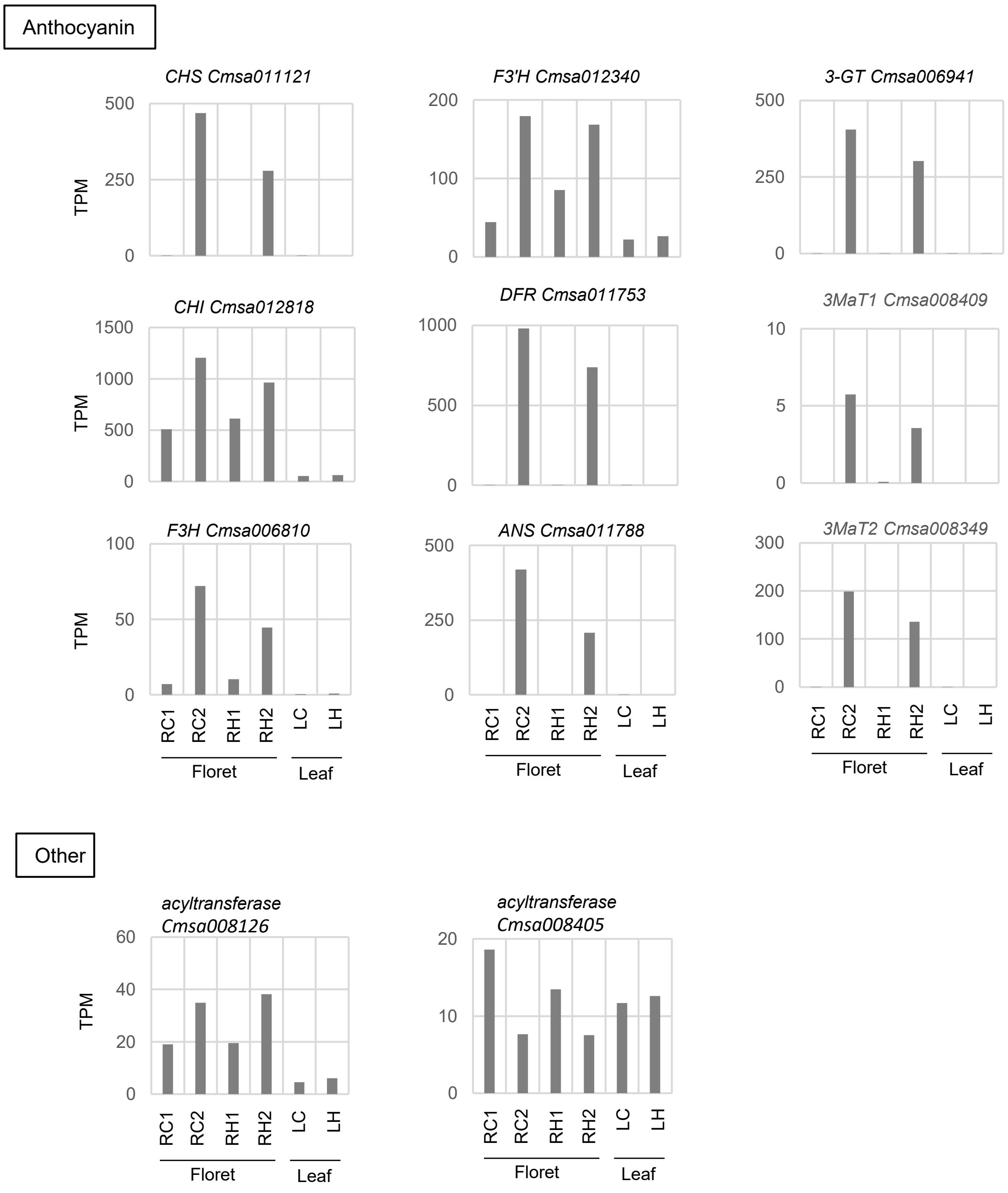

Figure S12. TPM values of the anthocyanin biosynthesis gene orthologs in each RNA-seq sample.

Each sample was mixed with three biological replicates. There was no replicate of each RNA-seq experiment. The vertical bar indicates the TPM value.

RC1: short-read sequences from ray florets grown under control conditions, stage 1. RC2: short-read sequences from ray florets grown under control conditions, stage 3. RH1: short-read sequences from ray florets grown at the high nighttime temperature, stage 1. RH2: short-read sequences from ray florets grown at the high nighttime temperature, stage 3. LC: short-read sequences from leaves grown under control conditions. LH: short-read sequences from leaves grown at the high nighttime temperature. The growth stages of ray-floret samples are indicated in Fig. 2.

References

Brugliera F, Holton TA, Stevenson TW, Farcy E, Lu C-Y, Cornish EC (1994) Isolation and characterization of a cDNA clone corresponding to the Rt locus of Petunia hybrida. Plant J 5:81–92. https://doi.org/10.1046/j.1365-313X.1994.5010081.x

Dong W, Li M, Li Z, Li S, Zhu Y, Hongxu, Wang Z (2020) Transcriptome analysis of the molecular mechanism of Chrysanthemum flower color change under short-day photoperiods. Plant Physiol Biochem 146:315–328. https://doi.org/10.1016/j.plaphy.2019.11.027

Hong Y, Tang X, Huang H, Zhang Y, Dai S (2015) Transcriptomic analyses reveal species-specific light-induced anthocyanin biosynthesis in chrysanthemum. BMC Genomics 16:202. https://doi.org/10.1186/s12864-015-1428-1

Lim S-H, Park B, Kim D-H, Park S, Yang J-H, Jung J-A, Lee J, Lee J-Y (2020) Cloning and Functional Characterization of Dihydroflavonol 4-Reductase Gene Involved in Anthocyanin Biosynthesis of Chrysanthemum. IJMS 21:7960. https://doi.org/10.3390/ijms21217960

Liu H, Luo C, Chen D, Wang Y, Guo S, Chen X, Bai J, Li M, Huang X, Cheng X, Huang C (2021) Whole-transcriptome analysis of differentially expressed genes in the mutant and normal capitula of Chrysanthemum morifolium. BMC Genom Data 22:2. https://doi.org/10.1186/s12863-021-00959-2

Liu H, Sun M, Du D, Pan H, Cheng T, Wang J, Zhang Q (2015) Whole-transcriptome analysis of differentially expressed genes in the vegetative buds, floral buds and buds of Chrysanthemum morifolium. PLoS ONE 10:e0128009. https://doi.org/10.1371/journal.pone.0128009

Ren L, Liu T, Cheng Y, Sun J, Gao J, Dong B, Chen S, Chen F, Jiang J (2016) Transcriptomic analysis of differentially expressed genes in the floral transition of the summer flowering chrysanthemum. BMC Genomics 17:673. https://doi.org/10.1186/s12864-016-3024-4

Sasaki K, Mitsuda N, Nashima K, Kishimoto K, Katayose Y, Kanamori H, Ohmiya A (2017) Generation of expressed sequence tags for discovery of genes responsible for floral traits of Chrysanthemum morifolium by next-generation sequencing technology. BMC Genomics 18:683. https://doi.org/10.1186/s12864-017-4061-3

Unno H, Ichimaida F, Suzuki H, Takahashi S, Tanaka Y, Saito A, Nishino T, Kusunoki M, Nakayama T (2007) Structural and mutational studies of anthocyanin malonyltransferases establish the features of BAHD enzyme catalysis. J Biol Chem 282:15812–15822. https://doi.org/10.1074/jbc.M700638200

Wang J, Wang H, Ding L, Song A, Shen F, Jiang J, Chen S, Chen F (2017) Transcriptomic and hormone analyses reveal mechanisms underlying petal elongation in Chrysanthemum morifolium 'Jinba.' Plant Mol Biol 93:593–606. https://doi.org/10.1007/s11103-017-0584-x

Wang K, Bai Z, Liang Q, Liu Q, Zhang L, Pan Y, Liu G, Jiang B, Zhang F, Jia Y (2018) Transcriptome analysis of chrysanthemum (Dendranthema grandiflorum) in response to low temperature stress. BMC Genomics 19:319. https://doi.org/10.1186/s12864-018-4706-x

Won SY, Kwon S-J, Lee T-H, Jung J-A, Kim JS, Kang S-H, Sohn S-H (2017) Comparative transcriptome analysis reveals whole-genome duplications and gene selection patterns in cultivated and wild Chrysanthemum species. Plant Mol Biol 95:451–461. https://doi.org/10.1007/s11103-017-0663-z

Yue J, Zhu C, Zhou Y, Niu X, Miao M, Tang X, Chen F, Zhao W, Liu Y (2018) Transcriptome analysis of differentially expressed unigenes involved in flavonoid biosynthesis during flower development of Chrysanthemum morifolium 'Chuju.' Sci Rep 8:13414. https://doi.org/10.1038/s41598-018-31831-6

Zhang X, Sun X, Zhang S, Yang J, Liu F, Fan J (2019) Comprehensive transcriptome analysis of grafting onto Artemisia scoparia W. to affect the aphid resistance of chrysanthemum (Chrysanthemum morifolium T.). BMC Genomics 20:776. https://doi.org/10.1186/s12864-019-6158-3
